# Natural Variations of *ZmRLR1* Mediate the Root Lodging Resistance of Maize by Regulating Root Ascorbate and Auxin Homeostasis

**DOI:** 10.1002/advs.202519638

**Published:** 2026-01-15

**Authors:** Wenshuai Lv, Pengshuai Yan, Huan Chen, Guangxian Li, Yiyue Zhao, Yanhui Chen, Wen‐Xue Li, Qingguo Du

**Affiliations:** ^1^ State Key Laboratory of Crop Gene Resources and Breeding National Engineering Laboratory For Crop Molecular Breeding Institute of Crop Sciences Chinese Academy of Agricultural Sciences Beijing China; ^2^ Seeds Research Syngenta Biotechnology China Beijing China

**Keywords:** ascorbate, clathrin‐mediated endocytosis, lodging, maize, root

## Abstract

Root lodging is a major problem limiting crop yield and seed quality. Here, we showed that *Root Lodging Resistance 1* (*ZmRLR1*) is specifically expressed in maize roots. The overexpression of *ZmRLR1* increased the rhizosphere soil weight, stem pulling force of the internode 50 cm from the ground, total root length, and root volume. In contrast, the rhizosphere soil weight, stem pulling force of the internode 50 cm from the ground, total root length, and root volume were reduced in the *Zmrlr1_ems_* and *Zmrlr1* mutants. Natural variations in the promoter of *ZmRLR1* are significantly associated with the root lodging resistance of maize. *ZmRLR1* positively regulates the ascorbate (AsA) content in maize roots. We further demonstrated that ZmRLR1 improved the interaction between the ZmAP2 σ subunit and ZmCHC2. The internalization of the endocytic tracer FM4‐64 is substantially reduced in the *Zmrlr1_ems_* and *Zmrlr1* mutants, while auxin distribution and ZmPIN1a‐YFP localization are altered in *Zmrlr1_ems_*. The defect in the total root length of *Zmrlr1_ems_* is rescued by the exogenous application of 10 µmol L^−1^ AsA plus 0.01 µmol L^−1^ 1‐naphthaleneacetic acid. Taken together, these results suggest that *ZmRLR1* affects the root lodging resistance of maize by regulating root AsA and auxin homeostasis.

## Graphical abstract

Root lodging severely limits crop yield and quality. We reveal that ZmRLR1, a plasma membrane b‐type cytochrome, regulates lodging resistance in maize through dual roles: modulating intracellular redox balance and functioning as a novel component of clathrin‐mediated endocytosis to influence auxin homeostasis. Moreover, natural variations in the *ZmRLR1* promoter affect the root lodging resistance of maize.

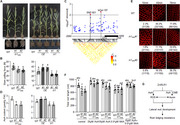

## 1 Introduction

By reducing the photosynthetic capacity, obstructing mechanical harvesting, and increasing the risk of ear rot, lodging has emerged as one of the primary challenges limiting cereal productivity worldwide [1, 2, 3]. In 2020, Typhoon Maysak caused severe lodging of maize across Northeast China [3]. In the same year, a derecho swept through the U.S. corn belt, damaging 8.2 million acres of maize in Iowa and leading to an economic loss of more than $7.5 billion [4]. To meet the demands of a growing world population, breeders tend to select high‐density tolerant maize varieties. However, root weight in both the horizontal and vertical directions decreases under high‐density conditions, which increases the risk of maize lodging [5, 6].

The lodging of cereal plants can be divided into stem lodging and root lodging. The former is defined as the buckling of the stem to the ground, whereas the latter is defined as the inability of the roots to provide adequate anchorage [7, 8]. Root lodging is typically induced by storms occurring during the jointing to grain‐filling stages of maize, potentially leading to yield losses of up to 30% [9]. Root system architecture (RSA), including root mass, volume, numbers, angle, and depth, is correlated with root lodging [10, 11]. Given that the morphological and anatomical characteristics influencing stem lodging could be readily characterized, identifying the genetic basis of stem lodging resistance in maize is feasible [12, 13, 14, 15, 16]. In contrast, a few studies have explored the genetic mechanisms responsible for root lodging resistance in maize, primarily because of the heavy workload, low‐throughput, and large artificial error.

The intricate root systems of maize include embryonic roots (primary roots and seminal roots) and postembryonic roots (nodal roots and lateral roots) [17]. Limited evidence suggests that the genetic improvement of RSA could increase the root lodging resistance of maize. The constitutive overexpression *ARGOS8*, a component of the ethylene receptor signaling complex, delayed the emergence of nodal roots and consequently reduced the root lodging resistance of maize [18]. YUCCA (YUC) encodes a rate‐limiting enzyme in auxin biosynthesis. Compared with wild‐type (WT) maize, the *Zmyuc4* single mutant and the *Zmyuc2/4* double mutant presented an enlarged brace root angle and increased resistance to lodging [19]. *ZmRSA3.1* encodes a member of the AUX/IAA proteins and contributes to the regulation of the root angle and depth [20], suggesting its potential role in the root lodging resistance of maize. Nevertheless, a comprehensive understanding of the molecular mechanisms regulating root lodging resistance in maize remains elusive.

Clathrin‐mediated endocytosis (CME) is the major gateway for eukaryotic cells to internalize plasma membrane proteins, lipids, and extracellular materials into the endosome [21]. In plants, CME is involved in various developmental processes and stress responses, including gametophyte development [22], embryogenesis [23], nutrient uptake [24, 25], phytohormone signaling [26, 27], and plant‐microbe interactions [28, 29]. Clathrin is a triskelion‐shaped complex composed of three clathrin heavy chains (CHCs) and three clathrin light chains (CLCs). However, clathrin cannot directly bind to the plasma membrane or cargo; therefore, the formation of clathrin‐coated vesicles (CCVs) requires cargo proteins, adaptor protein complex 2 (AP2), and various accessory protein factors [30]. The presence of homologs for several CME components across plants, animals, and yeast indicates that many aspects of CME are evolutionarily conserved [31]. However, a significant number of adaptors and accessory proteins identified in animals or yeast are notably absent in plants [32]. Consequently, identifying novel components to elucidate the molecular pathways underlying CME in plants is imperative.

In the present study, we identified and characterized a root‐specific gene, *Root Lodging Resistance 1* (*ZmRLR1*). *ZmRLR1* positively regulates the root lodging resistance of maize by mediating lateral root development. As a member of the plasma membrane b‐type cytochrome family, *ZmRLR1* also affects ascorbate (AsA) content in maize roots. We further revealed that ZmRLR1 serves as a novel component in CME and enhances the interaction between the ZmAP2 σ subunit and ZmCHC2. *ZmRLR1* also affects the expression of genes involved in auxin‐related processes. Notably, the exogenous application of AsA or AsA combined with 1‐naphthaleneacetic acid (1‐NAA) rescues the phenotypic defects in the total root length of *Zmrlr1_ems_*. These findings reveal an important role of *ZmRLR1* in root lodging resistance of maize by the regulation of root AsA and auxin homeostasis.

## 2 Results

### 2.1 *ZmRLR1* Positively Regulates the Root Size and Root Lodging Resistance of Maize

**FIGURE 1:**
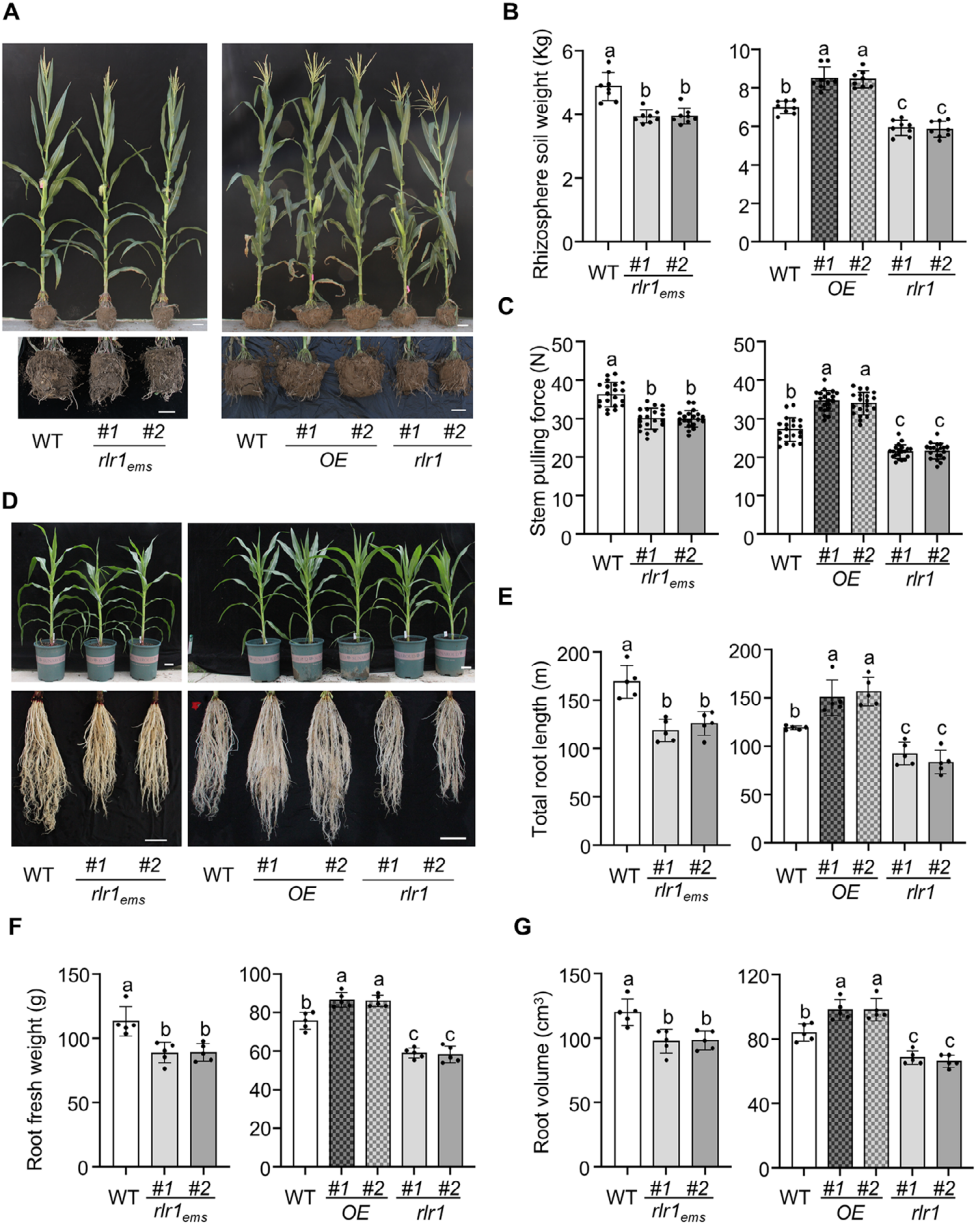
*ZmRLR1* positively regulates the root size and root lodging resistance of maize. (A) Effects of *ZmRLR1* expression on the growth of field‐grown maize. Representative images are shown. Scale bars = 10 cm. (B) Effects of *ZmRLR1* expression on the rhizosphere soil weight (*n* = 8). (C) Effects of *ZmRLR1* expression on the stem pulling force of the internode 50 cm from the ground of field‐grown maize (*n* = 20). (D) Effects of *ZmRLR1* expression on the growth of pot‐grown maize and root size. Representative images are shown. Scale bars = 10 cm. Effects of *ZmRLR1* expression on the total root length (E), root fresh weight (F), and root volume (G) of pot‐grown maize (*n* = 5). Means with the same letter are not significantly different at *p* < 0.05 according to one‐way ANOVA followed by Tukey's multiple comparison test.

To identify genes involved in the root lodging resistance of maize, we focused on genes that were specifically expressed in maize roots. The transcriptome profiles of various maize tissues were downloaded from the National Center for Biotechnology Information for analysis (http://www.ncbi.nlm.nih.gov) [33, 34]. Approximately 40 genes specifically expressed in roots were identified. *Zm00001d021017* attracted our attention because of its high abundance in roots (Figure S1A). This gene also maps to a previously reported genomic region associated with root architectural traits [35]. The specificity of its expression in roots was verified by reverse transcription quantitative polymerase chain reaction (RT‐qPCR) (Figure S1B). We then constructed transgenic maize lines overexpressing *Zm00001d021017* in the CAL50 background. Two transgenic lines (*OE#1* and *OE#2*) were chosen for further analysis (Figure S2A). Although overexpression of *Zm00001d021017* did not affect maize development, kernel width, kernel length, or hundred‐kernel weight (Figures S3 and S4), the rhizosphere soil weight was significantly greater in *Zm00001d021017*‐overexpressing transgenic maize than in WT plants (Figure 1A,B). We also evaluated root lodging resistance by measuring the stem pulling force of the internode 50 cm from the ground at a 15° inclination angle. Compared with that of WT maize, the stem pulling force of the internode 50 cm from the ground was significantly greater in *Zm00001d021017*‐overexpressing maize (Figure 1C). These results indicate that *Zm00001d021017* is involved in the root lodging resistance of maize and has been designated as *ZmRLR1*.

Changes in root morphology are associated with the root lodging resistance of maize [36, 37, 38]. To accurately observe the root morphology, we cultivated *ZmRLR1*‐overexpressing transgenic maize in 21‐L pots and assessed their root traits at the V12 stage. The total root length in *ZmRLR1*‐overexpressing transgenic maize ranged from 150.38 to 156.58 m, which was approximately 1.3 times greater than that of WT plants. Additionally, both root volume and root fresh weight were significantly higher in *ZmRLR1*‐overexpressing transgenic maize than in WT plants (Figure 1D–G).

To further characterize the functions of *ZmRLR1* in the root lodging resistance of maize, we searched publicly available EMS mutant collections and obtained two EMS mutants of *ZmRLR1* in the B73 background from the Maize EMS‐induced Mutant Database (http://elabcaas.cn/memd/public/index.html#/, mutant IDs: EMS4‐292f33 and EMS4‐09aeed) [39]. The mutants EMS4‐292f33 and EMS4‐09aeed contained a G/A or a C/T substitution in the exon of *ZmRLR1*, respectively. These substitutions led to premature stop codons in the gene (Figure S2B). We designated the mutants as *Zmrlr1_ems_#1* and *Zmrlr1_ems_#2*. Although they affected maize height, mutations in *Zmrlr1_ems_* did not affect the internode number, days to anthesis, or kernel traits (Figures S3 and S4). However, we observed reductions in rhizosphere soil weight, the stem pulling force of the internode 50 cm from the ground, total root length, root volume, and root fresh weight in the *Zmrlr1_ems_* mutants (Figure 1A–G).

We also generated *ZmRLR1* null mutants (*Zmrlr1*) in the CAL50 background with CRISPR‐associated protein 9 (CRISPR/Cas9). Two guide RNAs were designed to target sequences at nucleotides 185–204 and 265–284 downstream of the ATG codon (Figure S2C). The *Zmrlr1* mutants presented deletions of 79 or 80‐bp fragments in the coding sequence of *ZmRLR1*, which resulted in frameshifts (Figure S2C). Consistent with the observations in *Zmrlr1_ems_*, the *Zmrlr1* mutants presented reductions in rhizosphere soil weight, the stem pulling force of the internode 50 cm from the ground, total root length, root volume, and root fresh weight (Figure 1A–G).

The results presented to this point were obtained from *Zmrlr1_ems_*, *Zmrlr1* mutants, and *ZmRLR1*‐overexpressing transgenic maize grown in Nankou, Beijing. The *Zmrlr1_ems_*, *Zmrlr1* mutants, and *ZmRLR1*‐overexpressing transgenic maize were also grown in Sanya, Hainan Province. Compared with those of WT maize, the rhizosphere soil weight and stem pulling force were lower in *Zmrlr1_ems_* and *Zmrlr1* mutants and greater in *ZmRLR1*‐overexpressing transgenic maize grown in Sanya, Hainan Province (Figure S5). These results demonstrated that *ZmRLR1* regulates root size and the stem pulling force in maize.

### 2.2 Natural Variations in the *ZmRLR1* Promoter Affect the Root Lodging Resistance of Maize

**FIGURE 2:**
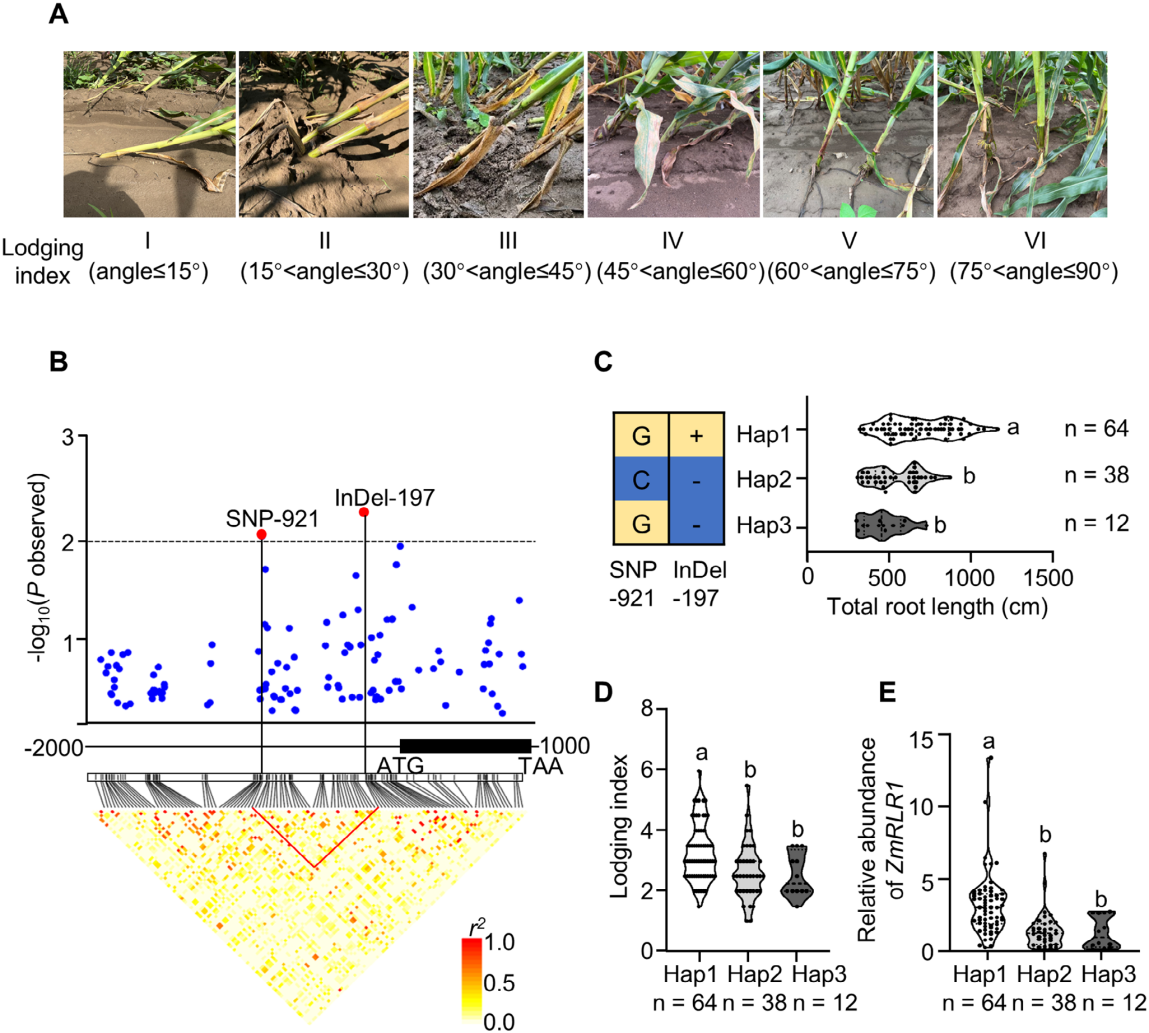
Natural variations in the *ZmRLR1* promoter are associated with root lodging resistance of maize. (A) Classification of the root lodging resistance of maize based on the angle between the stem and the ground. The numbers below the photographs indicate the lodging index. (B) *ZmRLR1*‐based association analysis and pairwise linkage disequilibrium analysis. Each dot represents a polymorphic single‐nucleotide polymorphism or an insertion‐deletion. A diagram of *ZmRLR1* is presented on the x‐axis. Identification of *ZmRLR1* haplotypes (Hap) for the total root length (C) and the root lodging index (D) in a natural maize population. *n* = 114 inbred lines. Each haplotype is displayed in a box plot. (E) Relative expression levels of *ZmRLR1* in each haplotype. Means with the same letter are not significantly different at *p* < 0.05 according to one‐way ANOVA followed by Tukey's multiple comparison test.

At midnight on July 26, 2022, a violent rainstorm struck the area of the Shunyi Agriculture Experimental Station of the Institute of Crop Sciences, Chinese Academy of Agricultural Sciences (Beijing). To assess lodging events in maize, the degree of root lodging of 122 inbred lines was immediately investigated the following day. The inbred lines were divided into six categories on the basis of the angle between the stem and the ground (Figure 2A). The total root length was positively correlated with the lodging index (Figure S6). These results further indicate that the total root length is a major factor affecting the root lodging resistance of maize.

We then re‐sequenced the promoter (2000‐bp region upstream of ATG) and the coding region of *ZmRLR1* in these inbred lines to investigate whether variations in *ZmRLR1* affect the root lodging resistance of maize. Candidate gene association analysis revealed that single nucleotide polymorphisms (SNP)‐921 and insertion‐deletion (InDel)‐197 in the *ZmRLR1* promoter were significantly associated with the lodging index (Figure 2B). These two SNPs accounted for 6.0% and 6.6% of the variation in the lodging index, respectively. The inbred lines were grouped into three principal haplotypes on the basis of the two variants (Hap1, Hap2, and Hap3) (Figure 2C). Compared with the inbred lines in Hap2 and Hap3, those in Hap1 were characterized by longer total root length, increased resistance to root lodging, and greater mRNA abundance of *ZmRLR1* (Figure 2C–E).

### 2.3 *ZmRLR1* Regulates Lateral Root Development in Maize

**FIGURE 3:**
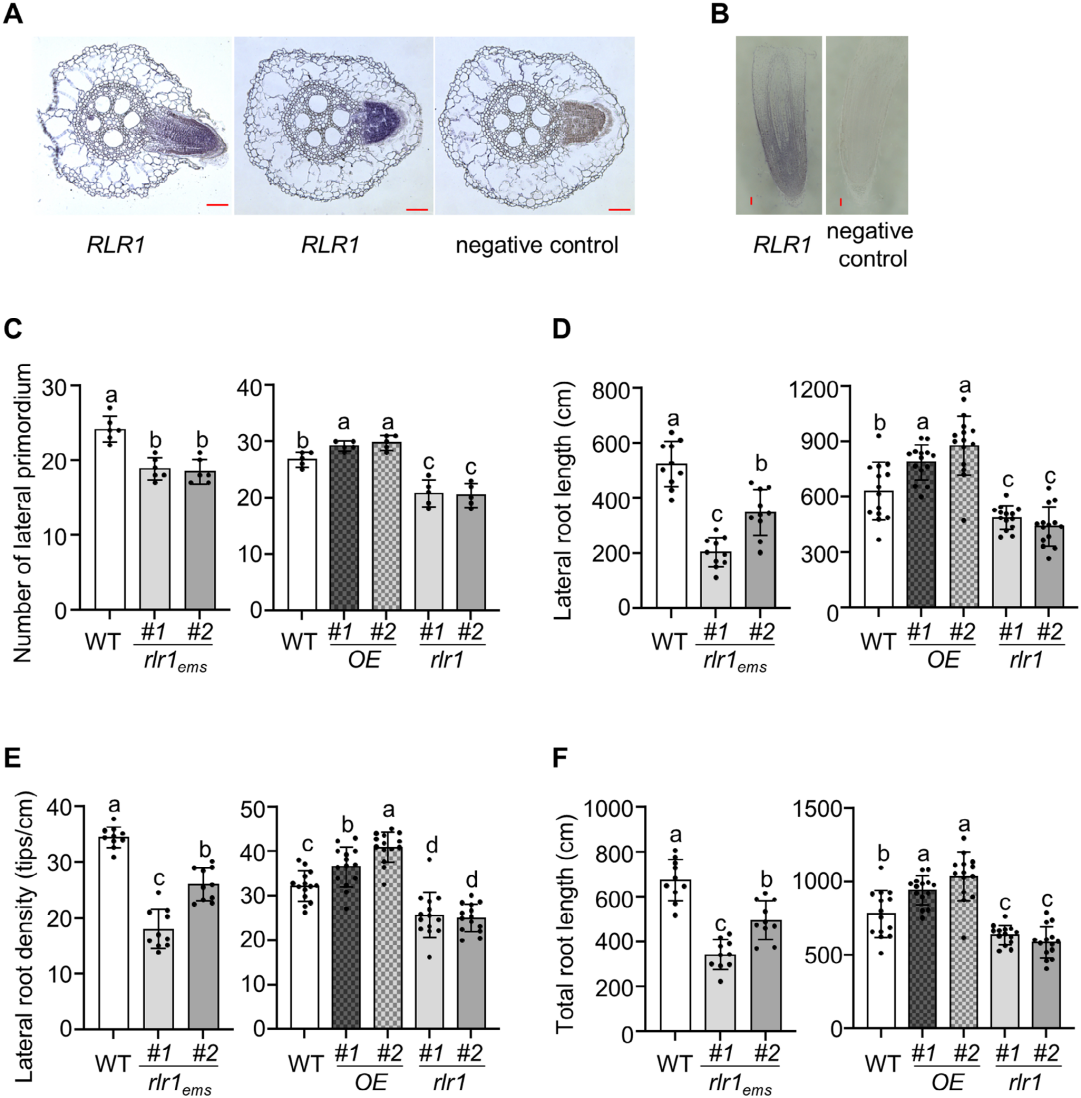
*ZmRLR1* positively regulates lateral root development in maize seedlings. Expression patterns of *ZmRLR1* in lateral roots (A) and primary roots (B) as determined by in situ hybridization analysis. Scale bars = 100 µm. (C) Effects of *ZmRLR1* expression on the number of lateral primordia in the seminal roots of maize (*n* = 5‐6). Effects of *ZmRLR1* expression on the lateral root length (D), lateral root density (E), and total root length of maize (F) (*n* = 10‐14). Means with the same letter are not significantly different at *p* < 0.05 according to one‐way ANOVA followed by Tukey's multiple comparison test.

To further explore the relationship between *ZmRLR1* and the root lodging resistance of maize, we first examined the expression patterns of *ZmRLR1* by in situ hybridization. *ZmRLR1* was detected in the lateral root, emerging lateral root, and primary root tip (Figure 3A,B). We germinated the seeds of WT, *Zmrlr1_ems_*, *Zmrlr1* mutants, and *ZmRLR1*‐overexpressing transgenic maize on paper rolls for 3 days. Seminal roots lacking lateral root emergence were sampled and stained with methylene blue to observe lateral root primordia. The overexpression of *ZmRLR1* significantly increased the number of lateral root primordia, whereas both the stop‐gain mutation in *ZmRLR1* and the loss‐of‐function mutation markedly reduced the number of lateral root primordia in maize (Figure 3C). These results suggested that *ZmRLR1* affects maize lateral root initiation.

We also cultivated WT, *Zmrlr1_ems_*, *Zmrlr1* mutants, and *ZmRLR1*‐overexpressing transgenic maize in hydroponic solution for 7 days. *ZmRLR1* expression did not affect the length of the primary root in maize (Figure S7). Compared with WT plants, *ZmRLR1*‐overexpressing transgenic maize presented significantly greater lateral root length, lateral root density, and total root length, whereas *Zmrlr1_ems_* and *Zmrlr1* mutants presented significantly lower values of these parameters (Figure 3D–F). These results further demonstrated that *ZmRLR1* regulates the lateral root development of maize.

### 2.4 *ZmRLR1* Affects AsA Homeostasis in Maize Roots

**FIGURE 4:**
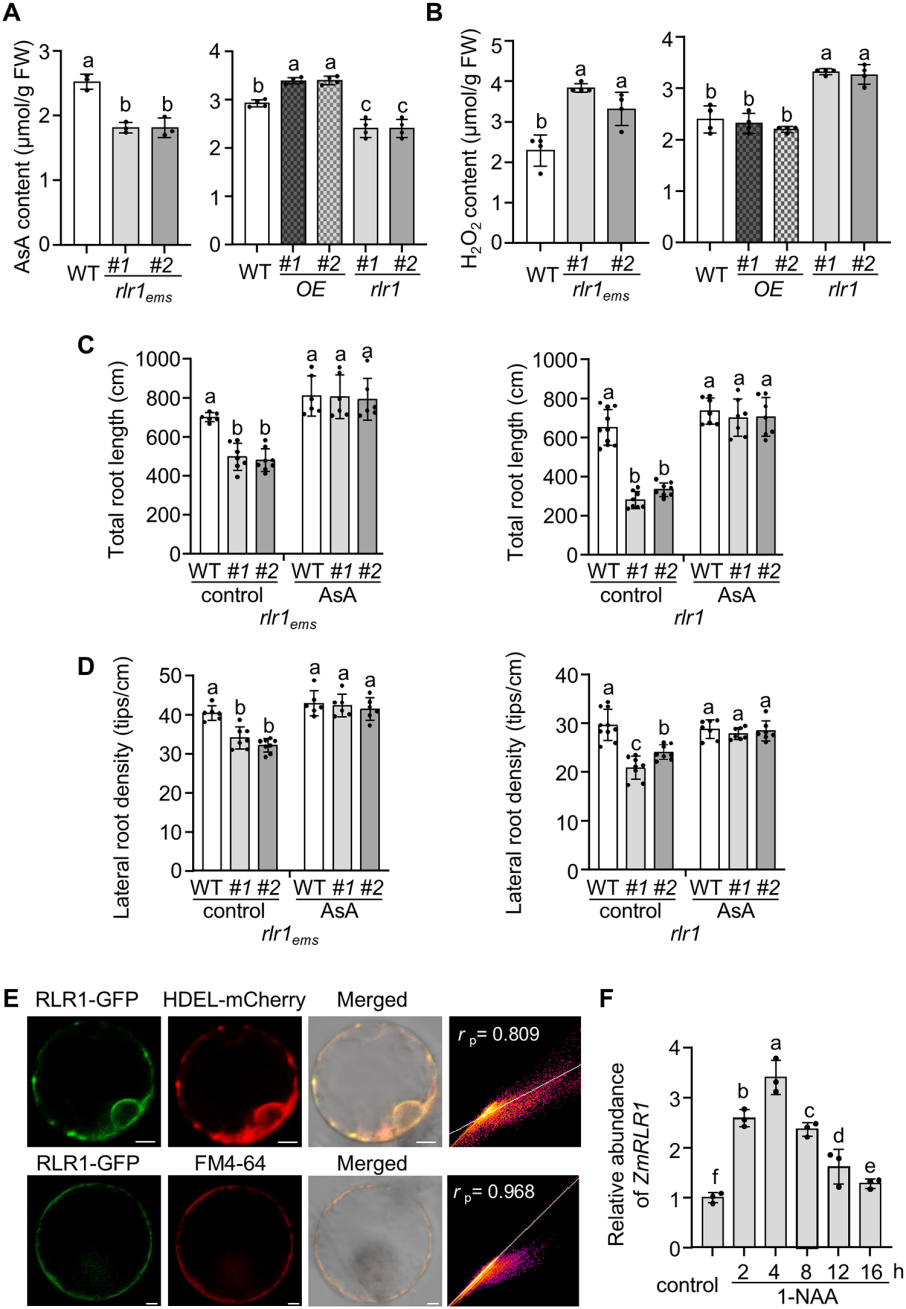
*ZmRLR1* affects ascorbate (AsA) and H_2_O_2_ contents in maize roots. (A) Effects of *ZmRLR1* expression on AsA levels in maize roots (*n* = 3‐4). (B) Effects of *ZmRLR1* expression on the H_2_O_2_ contents in maize roots (*n* = 4). The total root length (C) and lateral root density (D) of *Zmrlr1_ems_* and *Zmrlr1* mutants after being subjected to 25 µM AsA for 5 days (*n* = 6–10). (E) Subcellular localization of the ZmRLR1 protein. HDEL‐mCherry and FM4‐64 were used as endoplasmic reticulum and plasma membrane markers, respectively. Scale bars = 5 µm. (F) Effects of 1‐NAA on the mRNA abundance of *ZmRLR1*. The data were normalized to the expression of *ZmACTIN1*. The error bars represent the standard deviations of three biological replicates. Means with the same letter are not significantly different at *p* < 0.05 according to one‐way ANOVA followed by Tukey's multiple comparison test.

ZmRLR1 contains a dopamine *β*‐monooxygenase N terminus (DOMON) domain and belongs to a family of plasma membrane b‐type cytochromes that are specific to flowering plants [40]. *Arabidopsis AIR12* was the first member of this family identified. *AtAIR12* is involved in extracellular redox processes and can be fully reduced by AsA [40, 41]. We thus investigated the effects of *ZmRLR1* expression on AsA and H_2_O_2_ contents in maize roots. The overexpression of *ZmRLR1* significantly increased the AsA content in maize roots (Figure 4A). In contrast, stop‐gain or loss‐of‐function mutations in *ZmRLR1* resulted in decreased AsA levels and increased H_2_O_2_ contents in maize roots (Figure 4A,B).

We attempted to rescue the defects in the lateral roots of *Zmrlr1_ems_* and *Zmrlr1* mutants by the exogenous application of AsA. Following a 5‐day treatment with 25 µmol L^−^^1^ AsA, the lateral root density and total root length of *Zmrlr1_ems_* and *Zmrlr1* mutants were comparable to those of WT maize (Figure 4C,D). These results demonstrated that the phenotypic defects in the *Zmrlr1_ems_* and *Zmrlr1* mutants could be effectively rescued through the exogenous application of AsA.

### 2.5 *ZmRLR1* Presents Distinct Functions Compared to *AtAIR12*

Among all 11 genes encoding DOMON‐containing proteins in *Arabidopsis*, 10 are CYBDOMs (cytochrome b561 and DOMON domain), whereas *AIR12* encodes a protein constituted by a single DOMON associated with a glycosylphosphatidylinositol (GPI) membrane anchor [40]. When green fluorescent protein (GFP) was fused to the C‐terminus of AtAIR12 and transiently expressed in *Arabidopsis* mesophyll protoplasts, AtAIR12‐GFP signals were detected in both the plasma membrane and the endoplasmic reticulum (Figure S8). ZmRLR1 also encodes a DOMON protein associated with a GPI membrane anchor (Figure S9). When expressed in maize root protoplasts, ZmRLR1 was also localized to the plasma membrane and the endoplasmic reticulum (Figure 4E).

*AtAIR12* is an auxin‐inducible gene [42]. We thus investigated the responses of *ZmRLR1* to auxin. The exogenous application of the active auxin compound 1‐NAA, but not the inactive auxin compound 2‐NAA, rapidly induced *ZmRLR1* expression (Figure 4F; Figure S10). The mRNA abundance of *ZmRLR1* peaked after 4 h of 1‐NAA treatment and then gradually decreased (Figure 4F). This pattern is different from the continuous induction of *AtAIR12* by 1‐NAA in *Arabidopsis* [43].

Disruption of *AtAIR12* affects primary and lateral root development in *Arabidopsis* [44]. To further investigate the functions of *ZmRLR1* in root development, we overexpressed *ZmRLR1* in the *Atair12* mutant under the control of the constitutive *CaMV 35S* promoter (Figure S11). The overexpression of *ZmRLR1* rescued the decrease in the number of lateral root number of *Atair12*, but it did not rescue the shortened primary roots. These results suggest that *ZmRLR1* and *AtAIR12* have at least partially different functions.

### 2.6 ZmRLR1 Improves the Interaction Between the ZmAP2 σ subunit and ZmCHC2

**FIGURE 5:**
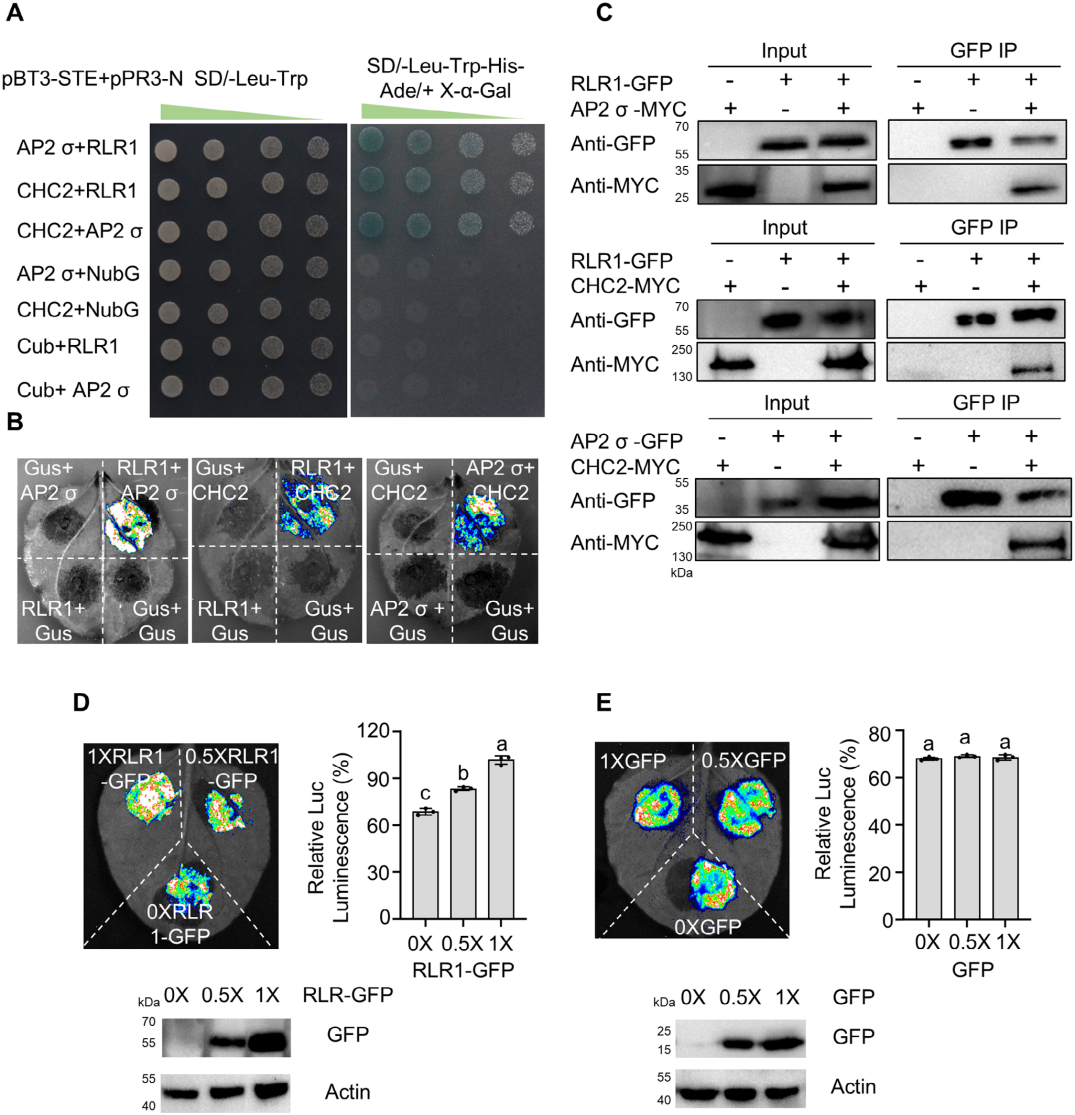
ZmRLR1 improves the interaction between the ZmAP2 σ subunit and ZmCHC2. Interactions between ZmRLR1 and the ZmAP2 σ subunit, ZmRLR1 and ZmCHC2, and the ZmAP2 σ subunit and ZmCHC2 in yeast and plants, as indicated by split‐ubiquitin yeast two‐hybrid assays (A), split firefly luciferase complementation assays in *N. benthamiana* leaves (B), and in vivo co‐immunoprecipitation assay in *N. benthamiana* leaves (C). Representative photographs are shown. Interactions among ZmRLR1/ZmAP2 σ subunit/ZmCHC2 (D) and GFP/ZmAP2 σ subunit/ZmCHC2 (E), as indicated by the split firefly luciferase complementation assay in *N. benthamiana* leaves. The input amounts of *ZmRLR1*‐GFP or GFP were detected using anti‐GFP antiserum diluted 1:5000. Actin was used as the loading control. Relative Luc luminescence is represented by the gray values calculated by ImageJ. The error bars represent the standard deviations of three biological replicates. Means with the same letter in (D) and (E) are not significantly different at *p* < 0.05 according to one‐way ANOVA followed by Tukey's multiple comparison test.

To test whether *ZmRLR1* utilizes additional molecular pathways beyond AsA homeostasis to affect lateral root development in maize, a pull‐down coupled with a liquid chromatography‐mass spectrometry (LC‐MS) assay was performed to identify proteins that potentially interacted with ZmRLR1 via membrane proteins isolated from the roots of the inbred line B73 at the V3 stage. A total of 39 proteins were identified as potential interactors of ZmRLR1 (Table S1). Among these, ZmCHC2 (Zm00001d005090) and the ZmAP2 σ subunit (Zm00001d041778) attracted our attention, as both are critical components of CME. The interactions between ZmRLR1 and the ZmAP2 σ subunit, ZmRLR1 and ZmCHC2, and the ZmAP2 σ subunit and ZmCHC2 were verified by a split‐ubiquitin membrane‐based yeast two‐hybrid system, a firefly luciferase complementation imaging assay, bimolecular fluorescence complementation (BiFC) analysis, and co‐immunoprecipitation (Co‐IP). The interaction resulted in survival on selective medium [synthetic defined (SD)/‐Leu/‐Trp/‐His/‐Ade] along with *α*‐galactosidase activity, strong luciferase (LUC) activity in tobacco leaves following the addition of luciferin (the substrate for firefly LUC), fluorescence reconstitution in maize mesophyll protoplasts, or Co‐IP of targeted proteins (Figure 5A–C; Figure S12). These results suggested that ZmRLR1 might represent a novel component of CME and might modulate the interaction between the ZmAP2 σ subunit and ZmCHC2.

To test this hypothesis further, ZmRLR1 or GFP was coexpressed with the ZmAP2 σ subunit and ZmCHC2. Western blot analysis was used to confirm the expression of ZmRLR1 or GFP in *Nicotiana benthamiana* leaves (Figure 5D,E). LUC activity was significantly greater in the ZmRLR1/ZmAP2 σ subunit/ZmCHC2 combination than in the ZmAP2 σ subunit/ZmCHC2 combination, with the activity increasing in proportion to the amount of ZmRLR1 added (Figure 5D). In contrast, compared with the ZmAP2 σ subunit/ZmCHC2 combination, the coexpression of GFP with the ZmAP2 σ subunit and ZmCHC2 combination did not significantly affect LUC activity (Figure 5E). These results indicate that ZmRLR1 enhances the interaction between the ZmAP2 σ subunit and ZmCHC2.

### 2.7 The *Zmrlr1_ems_* and *Zmrlr1* Mutants are Defective in Endocytosis

**FIGURE 6:**
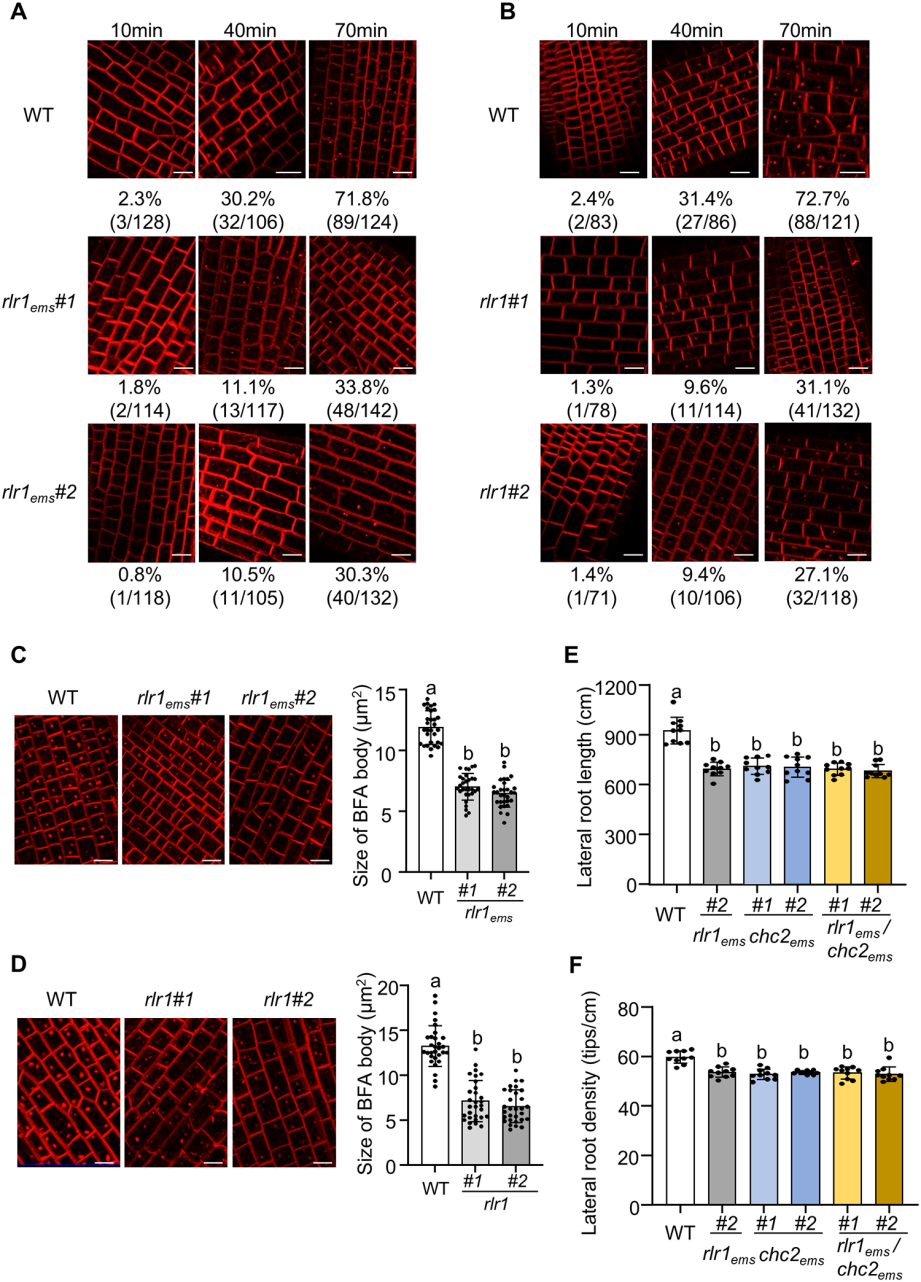
*ZmRLR1* loss of function delayed endocytosis of FM4‐64. Three‐dimensional reconstructions of z‐stacks were obtained for the *Zmrlr1_ems_* (A) and the *Zmrlr1* mutants (B). Boxplots of FM4‐64‐labeled BFA bodies in the *Zmrlr1_ems_* (C) and the *Zmrlr1* mutants (D). Lateral root length (E) and lateral root density (F) of *Zmrlr1_ems_*, *Zmchc2_ems_*, and *Zmrlr1_ems_*/*Zmchc2_ems_* in hydroponic solution for 10 days. Representative photographs at the indicated durations are shown. The numbers below the photographs indicate the percentage of FM4‐64‐labeled fluorescent puncta (% and number of FM4‐64‐labeled fluorescent puncta/total cell number). Scale bars = 20 µm. The error bars represent the standard deviation (*n* = 30 in (C) and (D), *n* = 10 in (E) and (F)). Means with the same letter are not significantly different at *p* < 0.05 according to one‐way ANOVA followed by Tukey's multiple comparison test.

To determine whether ZmRLR1 is involved in CME in maize, the uptake of N‐(3‐triethylammonium‐propyl)‐4‐(4‐diethylaminophenylhexatrienyl) pyridinium dibromide (FM4‐64) was examined in *Zmrlr1_ems_* and *Zmrlr1* mutants. Owing to the considerable size of the maize seedlings, comparable root sections from the *Zmrlr1_ems_*, *Zmrlr1* mutants and their corresponding WT seedlings were used to monitor FM4‐64 endocytosis [45]. After labeling for 40 min, FM4‐64‐labeled fluorescent puncta were detected in 30.2% of the WT root cells (32 of 106 cells), whereas only 10.5% to 11.1% of *Zmrlr1_ems_* root cells presented labeled puncta (11 of 105 cells or 13 of 117 cells) (Figure 6A). After labeling for 70 min, FM4‐64‐labeled fluorescent puncta were observed in only 30.3% to 33.8% of *Zmrlr1_ems_* root cells (40 of 132 cells or 48 of 142 cells), and in 71.8% of the WT root cells (89 of 124 cells) (Figure 6A). The effects of ZmRLR1 on endocytosis were further substantiated by the delayed internalization of FM4‐64 in *Zmrlr1* mutants (Figure 6B).

To further explore whether ZmRLR1 is a novel component of CME, we inhibited the endocytic recycling of FM4‐64 using the fungal toxin brefeldin A (BFA). The accumulation of FM4‐64 in BFA bodies was clearly observed in WT maize treated with BFA (Figure 6C,D). In contrast, the aggregation of FM4‐64 in BFA bodies was significantly reduced in *Zmrlr1_ems_* and *Zmrlr1* mutants (Figure 6C,D). Compared with those in WT plants, the sizes of FM4‐64‐labeled BFA bodies in *Zmrlr1_ems_* and *Zmrlr1* mutants decreased by ∼55% and 60%, respectively (Figure 6C,D). These results suggest that ZmRLR1 is important for endocytic transport.

We searched the publicly available EMS mutant collections and obtained two EMS mutants of *ZmCHC2* in the B73 background from the Maize EMS‐induced Mutant Database (mutant IDs: EMS4‐1ead2f and EMS4‐0f3304). The substitution in the exon of *ZmCHC2* led to a premature stop codon in this gene (Figure S13). The stop‐gain *ZmCHC2* mutant significantly reduced lateral root length and lateral root density but did not affect the length of primary roots in maize (Figure 6E,F; Figure S13). We then crossed *Zmrlr1_ems_#2* with *Zmchc2_ems_#1* or *Zmchc2_ems_#2*. The lateral root length and lateral root density in *Zmrlr1_ems_/Zmchc2_ems_* maize were comparable to those in *Zmrlr1_ems_#2* or *Zmchc2_ems_* plants (Figure 6E,F; Figure S14). These results suggested that *ZmRLR1* and *ZmCHC2* are located in the same pathway that regulates lateral root development in maize.

### 2.8 *ZmRLR1* Mediates Auxin Homeostasis in Maize Roots

**FIGURE 7:**
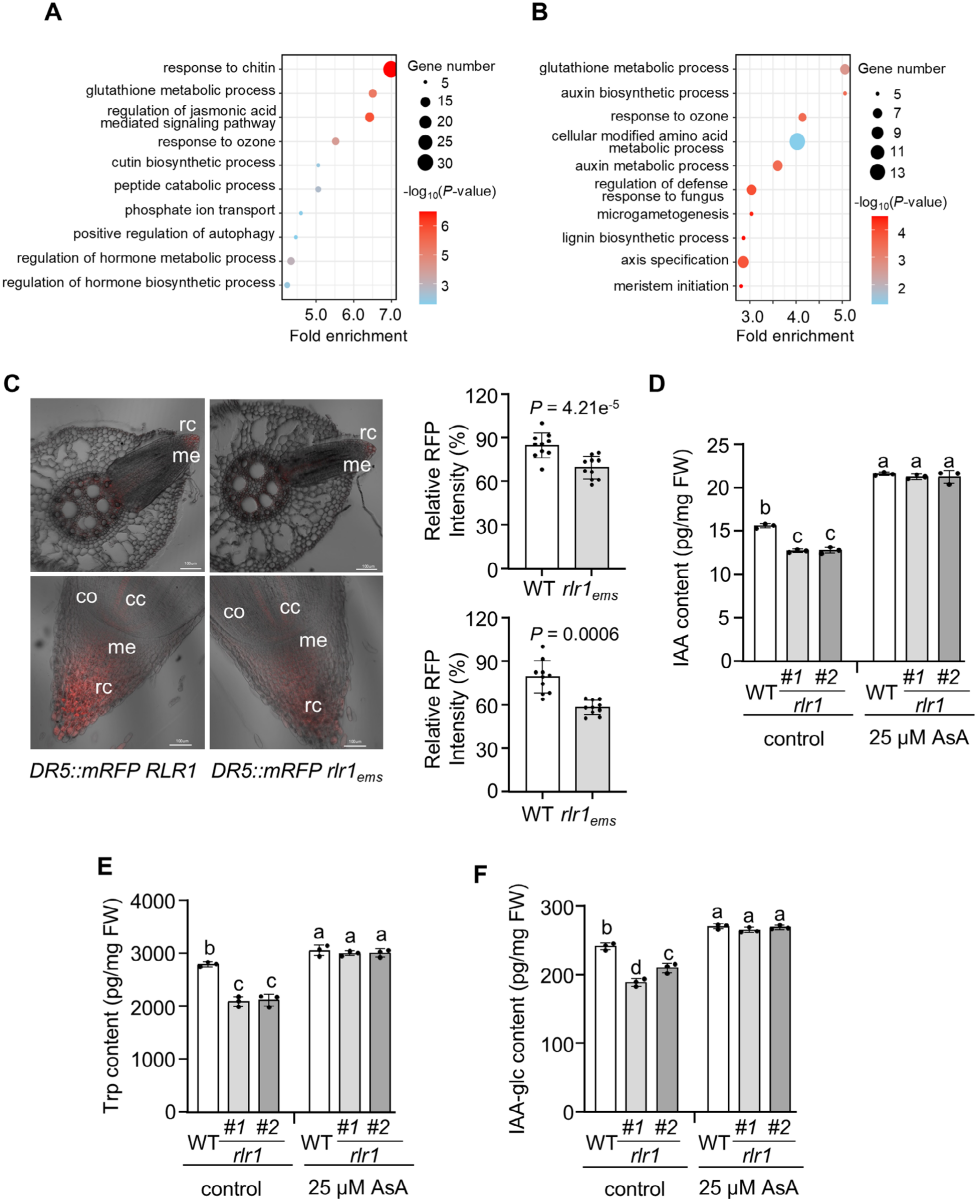
*ZmRLR1* regulates auxin homeostasis in maize roots. Gene Ontology analysis of differentially expressed genes in *Zmrlr1* mutant (A) and *ZmRLR1*‐overexpressing transgenic maize (B) compared with WT. The point size represents the number of genes in the pathway. The color depth indicates ‐log_10_(*p*‐value). (C) Fluorescence of *ZmDR5::mRFP* in the lateral roots (upper) and primary roots (lower) of WT maize and *Zmrlr1_ems_*. The relative RFP intensity is represented by the gray value calculated by ImageJ ( m *n* = 10). Scale bars = 100 µm. Free IAA content (D), Trp content (E), and IAA‐glc content (F) in the roots of WT maize and *Zmrlr1* mutants after exposure to 25 µm AsA for 5 days. Error bars represent the standard deviations of three biological replicates. The *p*‐values in (C) are determined using a two‐tailed unpaired *t* test. Means with the same letter in (D) to (F) are not significantly different at *p* < 0.05 according to one‐way ANOVA followed by Tukey's multiple comparison test. cc, central cylinder; me, root meristem; rc, root cap; co, cortex.

To further investigate the roles of *ZmRLR1* in maize root development, we compared the whole‐transcriptome profiles of the roots of WT, *Zmrlr1* mutant (#1), and *ZmRLR1*‐overexpressing transgenic maize (#1) grown in hydroponic solution for 7 days. Each sample included three biological replicates. Approximately 0.33 billion reads were perfectly mapped to maize B73_RefGen_v4. The Pearson correlation coefficient for all comparisons exceeded 0.98 (Figure S15), indicating a high correlation among the biological replicates. On the basis of the criteria of a log_2_ fold‐change ratio of ≥ 1 and a *p* value of ≤ 0.05, 1521 and 1598 genes, including *ZmRLR1* itself, were identified as differentially expressed genes (DEGs) in *Zmrlr1* mutant and *ZmRLR1*‐overexpressing transgenic maize, respectively. Gene Ontology (GO) analysis revealed that the 1521 DEGs in the *Zmrlr1* mutant were enriched for biological processes related to positive regulation of autophagy (GO:00 10508, *p* = 4.8e^−3^), regulation of hormone metabolic process (GO:00 32350, *p* = 4.62e^−4^), and regulation of hormone biosynthetic process (GO:00 46885, *p* = 2.68e^−3^) (Figure 7A). The 1598 DEGs in *ZmRLR1*‐overexpressing transgenic maize were enriched in biological processes associated with auxin biosynthetic process (GO:0 009851, *p* = 2.74e^−3^) and auxin metabolic process (GO:0 009850, *p* = 3.13e^−3^) (Figure 7B). These results indicated that *ZmRLR1* might affect auxin homeostasis in maize roots.

To test this hypothesis, we crossed auxin‐responsive *ZmDR5::RFP* reporter maize with *Zmrlr1_ems_*. Compared with those in WT maize, the RFP signals in the lateral roots and root caps of *Zmrlr1_ems_* were significantly lower (Figure 7C). We then measured the free IAA content in the roots of the *Zmrlr1* mutants. The free IAA content in the roots of the *Zmrlr1* mutants was approximately 12.8 pg mg^−1^ FW, which was significantly lower than that in WT maize (Figure 7D). The most comprehensively studied IAA biosynthetic pathways rely on Trp as a precursor [46]. The IAA‐glucose (IAA‐glc) conjugate is the most abundant inactive IAA form [47]. *ZmRLR1* loss‐of‐function mutations by CRISPR/Cas9 significantly reduced Trp and IAA‐glc concentrations in maize roots (Figure 7E,F). After 25 µm AsA treatment for 5 days, the concentrations of IAA, Trp, and IAA‐glc were significantly increased in *Zmrlr1* roots and no longer differed from those in WT maize treated under the same conditions (Figure 7D–F). These results further suggested that *ZmRLR1* affects IAA homeostasis in maize roots.

### 2.9 ZmPIN1a‐YFP localization in the *Zmrlr1_ems_* Mutant

**FIGURE 8:**
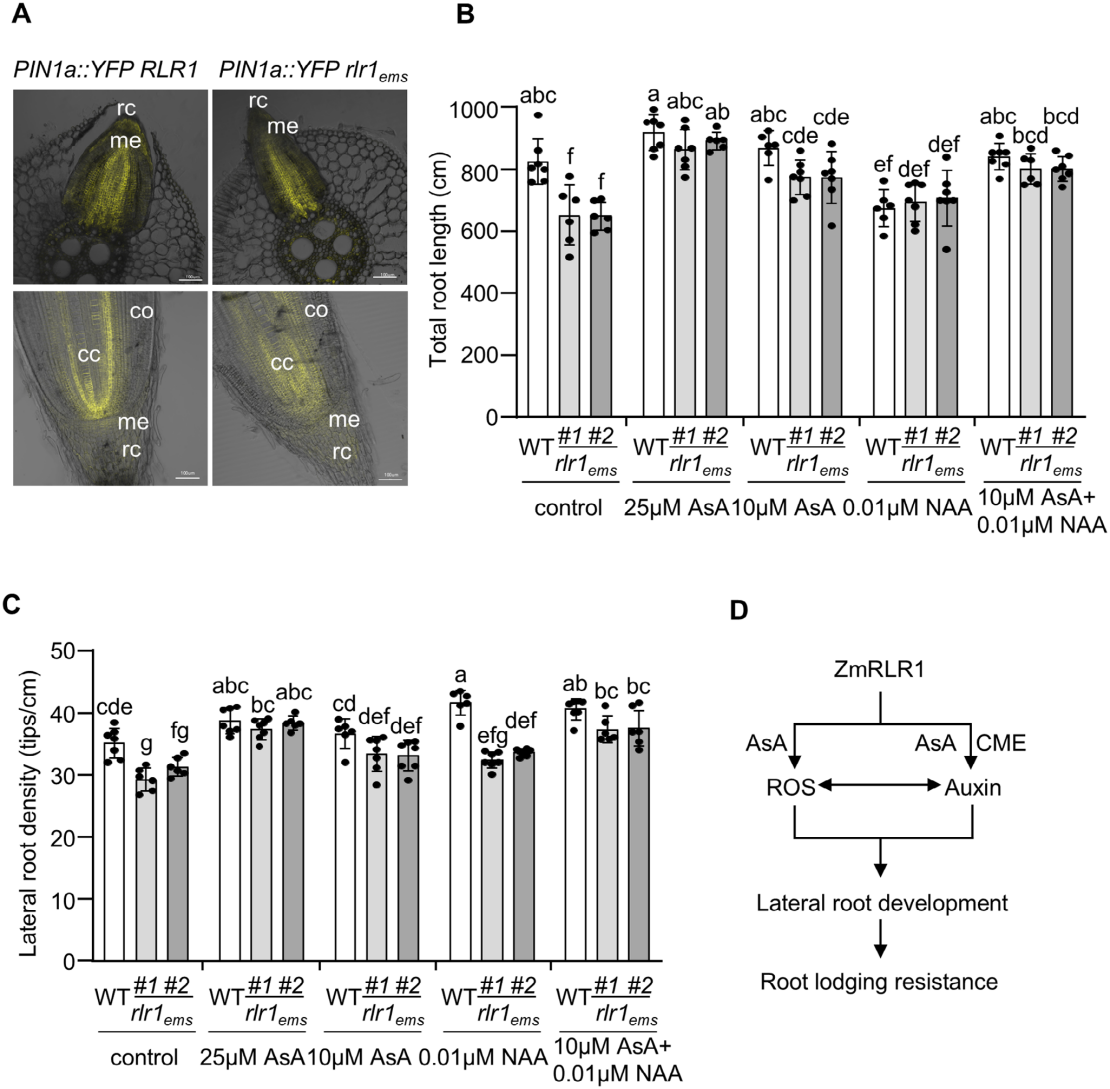
Both auxin and ascorbate (AsA) are involved in *ZmRLR1*‐mediated lateral root development in maize. (A) Fluorescence of *PIN1a::YFP* in the lateral roots (upper) and primary roots (lower) of WT maize and *Zmrlr1_ems_*. cc, central cylinder; me, root meristem; rc, root cap; co, cortex. Bars = 100 µm. Effects of various combinations of auxin and AsA on the total root length (B) and lateral root density (C) of WT maize and *Zmrlr1_ems_*. The error bars represent the standard deviation (*n* = 6‐7). Means with the same letter are not significantly different at *p* < 0.05 according to one‐way ANOVA followed by Tukey's multiple comparison test. (D) A proposed model for the role of *ZmRLR1* in maize root lodging resistance.

The PIN1 proteins are important for the polar transportation of auxin and affect plant root development [48, 49]. CME constitutes the predominant pathway for the internalization and recycling of PIN proteins [26]. To investigate the involvement of *ZmRLR1* in this process, we crossed the *ZmPIN1a*::*YFP* line with *Zmrlr1_ems_*. In WT maize, the YFP signals of PIN1a were detected mainly in the central cylinder of primary roots (Figure 8A). In contrast, the YFP signals in the primary roots of *Zmrlr1_ems_* were more diffuse than those in WT maize (Figure 8A). In the emerging lateral roots, the YFP signals observed in the root caps of WT maize were nearly absent in *Zmrlr1_ems_* (Figure 8A). These results further suggested that ZmRLR1 is involved in endocytic transport and could affect auxin homeostasis in maize roots.

To verify the roles of auxin and AsA in *ZmRLR1*‐mediated lateral root development in maize, we attempted to rescue the phenotypic defects of *Zmrlr1_ems_* by the exogenous application of 1‐NAA and AsA. Consistent with previous observations (Figure 4C,D), the phenotypic defects in the lateral root density and total root length of *Zmrlr1_ems_* were reversed by the exogenous application of 25 µmol L^−1^ AsA (Figure 8B,C). The application of 10 µmol L^−1^ AsA alleviated the phenotypic defects in lateral root density and total root length observed in *Zmrlr1_ems_* (Figure 8B,C). Moreover, the application of 0.01 µmol L^−1^ 1‐NAA promoted lateral root formation and inhibited the total root length of WT maize but had no significant effects on *Zmrlr1_ems_* (Figure 8B,C), suggesting that *Zmrlr1_ems_* is less sensitive to exogenous IAA treatment. However, the defect in the total root length of *Zmrlr1_ems_* was reversed by the combined exogenous application of 10 µmol L^−1^ AsA combined with 0.01 µmol L^−1^ 1‐NAA (Figure 8B).

## 3 Discussion

In the present study, we identified a root‐specific member of the plasma membrane b‐type cytochrome family, designated as *ZmRLR1*. Consistent with other members of this family, *ZmRLR1* affects AsA levels in maize roots. We further demonstrated that *ZmRLR1* mediates auxin homeostasis in maize roots by regulating CME. We thus hypothesized that *ZmRLR1* regulates the lateral root development and root lodging resistance of maize by concurrently regulating auxin and AsA homeostasis in maize roots (Figure 8D).

Although detailed information is available on the roles of endocytosis in animal cells, our understanding of endocytic mechanisms in plants remains limited [21]. Like animal cells, CCVs in plants are composed of CHCs, CLCs, and heterotetrameric AP complexes. The AP complexes facilitate the connection between clathrin and membrane‐associated lipids and/or cargo proteins at sites of CCV formation on the plasma membrane or at the trans‐Golgi network/early endosome [50]. On the basis of the direct interaction between the ZmRLR1 and the ZmAP2 σ subunit and between ZmRLR1 and ZmCHC2, as demonstrated by a split‐ubiquitin membrane‐based yeast two‐hybrid system, a firefly luciferase complementation imaging assay, BiFC analysis, and Co‐IP in the current study, we propose that ZmRLR1 is a novel component involved in CME through its interactions with the ZmAP2 σ subunit and ZmCHC2. This hypothesis was further supported by the delayed internalization of FM4‐64, the reduced sizes of FM4‐64‐labeled BFA bodies in *Zmrlr1_ems_* and *Zmrlr1* mutants, and the genetic relationship between *ZmRLR1* and *ZmCHC2*.

In *Arabidopsis*, AP2 σ subunit is primarily recruited from the cytoplasm to the plasma membrane to initiate CCV formation [23]. The AP2 σ subunit mutant displays reduced vegetative growth, impaired silique development, and drastically reduced fertility [23]. As a root‐specific gene, the overexpression or loss‐of‐function of *ZmRLR1* regulated the RSA and root lodging resistance of maize without affecting developmental stage or kernel traits. These results suggest that *ZmRLR1* could be a potential target for improving the root lodging resistance of maize through its role in mediating endocytosis.

The maize root system contains structurally and functionally different root types. Elements of the auxin‐mediated signaling pathway, including the LOB domain and Aux/IAA proteins, regulate the initiation of seminal, shoot‐borne, and lateral root [17]. *ZmRLR1* is located in the primary root tip, emerging lateral root, and lateral root of maize. The overexpression of *ZmRLR1* significantly increased the number of lateral root primordia, and the cessation of *ZmRLR1* or *ZmRLR1* loss‐of‐function markedly reduced the number of lateral root primordia. Furthermore, in the *Zmrlr1* mutants, the concentrations of IAA, Trp, and IAA‐glc were decreased and, after 5 days of AsA treatment, no longer differed from those in WT maize. The localization of ZmPIN1a was also altered in the lateral root and primary root tips of *Zmrlr1_ems_*. These results suggest that *ZmRLR1* mediates lateral root initiation by affecting local auxin homeostasis in maize roots.

Reactive oxygen species (ROS) are key elements determining root development in plants. Crosstalk between auxin and ROS is believed to control the growth and development of plant roots [51, 52, 53]. However, the molecular link between auxin and ROS remains to be explored. As a member of the plasma membrane b‐type cytochrome family, *ZmRLR1* affects AsA biosynthesis in maize. The application of 25 µmol L^−1^ AsA reversed the phenotypic defects of the *Zmrlr1_ems_* and *Zmrlr1* mutants. Additionally, *ZmRLR1* directly regulates auxin homeostasis in maize by mediating CME. The application of 0.01 µmol L^−1^ 1‐NAA inhibited the total root length of maize, and the inhibition was relieved by the application of 10 µmol L^−1^ AsA. When applied in conjunction with 1‐NAA, a lower concentration of AsA (10 µmol L^−1^) effectively rescued the phenotypic defects of *Zmrlr1_ems_*. These results suggest that *ZmRLR1* serves as a molecular link between auxin and ROS.

One challenge in developing maize varieties resistant to root lodging is the large‐scale assessment of the root lodging propensity of multiple inbred lines under field conditions. Here, we found: (1) overexpression of *ZmRLR1* increases the total root length, and *ZmRLR1*‐overexpressing transgenic maize is resistant to root lodging in the field; (2) loss‐of‐function of *ZmRLR1* reduces the total root length, and *Zmrlr1_ems_*/*Zmrlr1* mutants are sensitive to root lodging in the field; (3) the total root length is positively correlated with the root lodging index; and (4) the inbred lines in Hap1 are characterized by a longer total root length and greater resistance to root lodging. On the basis of these observations, we propose that the total root length could be a potential index for evaluating the root lodging resistance of maize.

## 4 Experimental Section

### 4.1 Plant Materials and Growth Conditions

Seeds were pretreated and germinated as previously described [13]. When the first leaf from the base of a seedling was fully expanded, the endosperm was removed. The seedlings were subsequently transferred to 12‐L pots filled with hydroponic solution. The plants were grown in half‐strength Hoagland's nutrient solution for 2 days, followed by an additional 5 days in full‐strength Hoagland's nutrient solution supplemented with either 25 µM AsA or 0.01 µM 1‐NAA. The plants were grown in a greenhouse with a 28/22°C day/night temperature regime and 14 h/10 h light/dark photoperiod.

In 2022, 122 maize inbred lines were planted at the Shunyi Agriculture Experimental Station of the Institute of Crop Sciences, Chinese Academy of Agricultural Sciences (116.65°E, 40.13°N). The experimental station was characterized by aquic cinnamon soil (pH 7.8). Transgenic maize was planted at the Nankou Experimental Station (Beijing; 116.12°E, 40.13°N), with sandy loam soil (pH 7.5) or at the Sanya Agriculture Experimental Station of the Institute of Crop Sciences, Chinese Academy of Agricultural Sciences (Hainan Province; 109.20°E, 18.40°N) with latosol soil (pH 4.9). The experimental design and planting pattern were performed as described by Guo et al. [3] The inbred lines used in this research are listed in Table S2.

*Arabidopsis thaliana* (L.) Heynh ecotype Columbia (Col‐0) was used for all the experiments. The T‐DNA insertion mutant of *AIR12* (SALK_134161) was kindly provided by Shaoyun Lu (South China Agricultural University) and Zhenfei Guo (Nanjing Agricultural University). *Arabidopsis* seedlings in Murashige and Skoog nutrient agar medium were grown under a 16‐h‐light/8‐h‐dark photoperiod at 23 ± 1°C.

### 4.2 Construction of Transgenic Maize

To generate CRISPR/Cas9‐mediated *ZmRLR1* loss of‐function mutants, two guide RNAs targeting sequences located at nucleotides 185–204 and 265–284 downstream of the ATG codon were designed. The fragments were subsequently cloned and inserted into the pBUE411 vector using the *Bsa*I restriction site by a T4 DNA ligase reaction.

To generate *ZmRLR1*‐overexpressing transgenic maize, the coding region of *ZmRLR1* was amplified from the inbred line B73 and cloned into the pCUB‐GFP‐MYC vector under the control of the maize ubiquitin promoter using the *Bst*EII restriction site by an In‐Fusion reaction. The verified vectors were subsequently electroporated into *Agrobacterium tumefaciens EHA105*, which was then transformed into immature embryos of the maize inbred line CAL50.

Transformants were selected with gradually increasing concentrations of bialaphos. For CRISPR/Cas9‐mediated *ZmRLR1* loss‐of‐function mutants, T_1_ or T_2_ homozygous lines were sequenced to ensure the knockout of *ZmRLR1*. Cas9‐free plants were identified for phenotypic analysis. For *ZmRLR1*‐overexpressing transgenic maize, T_2_ or T_3_ homozygous lines were used for the experiments. The sequences of the specific primers used are listed in Table S3.

### 4.3 Complementation of the *Atair12* Mutant

The full‐length CDS of *ZmRLR1* was amplified by PCR. The amplified fragment was subsequently cloned into the pCAMBIA1303 vector using the *Spe*I restriction site by an In‐Fusion reaction. The plasmids were electroporated into *Agrobacterium tumefaciens* GV3101 and transformed into the *Atair12* mutant by the floral dip method [54]. Transgenic plants were selected with 35 µg mL^−1^ hygromycin. T_2_ homozygous lines were used for the experiments. The sequences of the specific primers used are listed in Table S3.

### 4.4 Gene Expression Analysis

Total RNA extraction and purification, first‐strand cDNA synthesis, and real‐time quantitative RT‐PCR were performed as previously described [13]. Each analysis was based on three replications. The comparative Ct method was used for data analysis [55]. The sequences of the specific primers used are listed in Table S3.

### 4.5 Subcellular Localization of ZmRLR1

The coding region of *ZmRLR1* without a stop codon was amplified and cloned into the pEZS‐NL vector. A GFP was fused to the C‐terminus of ZmRLR1 using the *Eco*RI restriction site by an In‐Fusion reaction. The vector, along with an endoplasmic reticulum marker were transiently coexpressed in maize root protoplasts. The fluorescence images were collected with a Zeiss LSM700 confocal microscope. The sequences of the specific primers used are listed in Table S3.

### 4.6 in situ Hybridization

The B73 roots at the V3 stage were sampled for in situ hybridization. A specific 134‐bp fragment of *ZmRLR1* was amplified and cloned into the pGEM‐T‐easy vector (Promega, U.S.A.). Sense and antisense probes were generated by in vitro transcription using T7 or SP6 RNA polymerases. The probes were labeled with digoxigenin following the manufacturer's instructions (Roche, Switzerland). in situ hybridization and immunological detection were performed as previously described [13]. Images were collected with a Pannoramic MIDI microscope (3DHISTECH, Budapest, Hungary). The sequences of the specific primers used are listed in Table S3.

### 4.7 Split‐Ubiquitin Yeast Two‐Hybrid Assay

The DUALmembrane Kit (Dualsystems Biotech, Schlieren, Switzerland) was used for the yeast two‐hybrid assay. The coding regions of the *ZmAP2 σ* subunit, *ZmCHC2*, and *ZmRLR1* were amplified and cloned into the vectors pBT3‐STE and pPR3‐N NubG using the *Hind*III and *EcoR*I restriction sites by an In‐Fusion reaction. The primers used are listed in Table S3. Various combinations of *ZmAP2 σ* subunit, *ZmCHC2*, and *ZmRLR1* constructs were cotransformed into the yeast strain NMY51. The transformants were selected on synthetic medium lacking leucine (Leu) and tryptophan (Trp). The positive clones were plated on synthetic minimal medium without Leu, Trp, His, and Ade. Interaction was further confirmed by chloroform overlay *α*‐galactosidase plate assay without Leu, Trp, His, and Ade.

### 4.8 Firefly Luciferase Complementation Imaging Assay

The firefly luciferase complementation imaging assay was performed as described by Fàbregas et al. [56] The coding region of *ZmRLR1* was cloned into pCAMBIA1300‐nLUC and pCAMBIA1305‐GFP. The coding region of the *ZmAP2 σ* subunit was cloned into pCAMBIA1300‐nLUC and pCAMBIA1300‐cLUC. The coding region of *ZmCHC2* was cloned into pCAMBIA1300‐cLUC. Various combinations of constructs were transiently coexpressed in *N. benthamiana* leaves. After infiltration for 2 days, the luciferase signals were collected by a charge‐coupled device camera (Tanon‐5200, China). Each experiment was replicated three times. The gray values were quantified by ImageJ software.

### 4.9 Pull‐Down Plus LC‐MS Assays

Owing to the subcellular location of ZmRLR1 in the plasma membrane and endoplasmic reticulum, membrane proteins from the roots of the inbred line B73 at the V3 stage were used as input material to detect the proteins that interact with ZmRLR1. Both the GST and ZmRLR1‐GST proteins were expressed in the DE3 strain and subsequently bound to Glutathione Sepharose 4B agarose beads (GE Healthcare, U.S.A.) through ligand‐receptor interactions. The membrane proteins extracted from maize roots were incubated overnight with GST or ZmRLR1‐GST protein‐agarose bead complexes at 4°C. After electrophoresis in a denaturing polyacrylamide gel, staining was performed using the Pierce Silver Stain for mass spectrometry (Thermo Fisher, U.S.A.). The specific gel bands detected in the ZmRLR1‐GST sample were excised and analyzed by LC‐MS at the Mass Spectrum Laboratory of China Agricultural University. The sequences of the specific primers used are listed in Table S3.

### 4.10 Co‐IP Assays

Co‐IP was performed as described by Liu et al. [57] The coding regions of *ZmRLR1*, the *ZmAP2 σ* subunit, and *ZmCHC2* were cloned into pCAMBIA1305‐GFP or pSuper1300‐6MYC, respectively. Various combinations of constructs were transiently coexpressed in *N. benthamiana* leaves. After infiltration for 2 days, *N. benthamiana* leaves were homogenized in immunoprecipitation buffer containing 50 mm Tris‐HCl, pH 7.5, 150 mm NaCl, 1 mm EDTA, 1% Triton‐X100, 2 mm NaF, 2 mm Na_3_VO_4_, 3 mm dithiothreitol (DTT), and 1 × EDTA‐free proteinase inhibitor. After centrifugation, the supernatant was incubated with 30 uL of GFP‐Trap Magnetic Agarose (Chromo Tek, Germany) for 3 h at 4°C. The matrix beads were washed six times with immunoprecipitation buffer. The sequences of the specific primers used are listed in Table S3.

### 4.11 BiFC Assays

The coding sequences of *ZmRLR1*, the *ZmAP2 σ* subunit and *ZmCHC2* were amplified by the specific primers listed in Table S3. The amplified fragments were cloned into the pAN580‐nYFP or pAN580‐cYFP. Various combinations of the *ZmRLR1*, *ZmAP2 σ* subunit and *ZmCHC2* vectors were transiently expressed in maize mesophyll protoplasts. YFP images were obtained with a Zeiss LSM980 confocal microscope.

### 4.12 FM4‐64 Internalization Assay

To evaluate the rate of endocytosis, FM4‐64 internalization assays were carried out as previously described by Wang et al. [45] Maize seedlings were incubated in half‐strength Hoagland's nutrient solution supplemented with 5 µm FM4‐64 for 10 min. For BFA treatment, maize seedlings were incubated in half‐strength Hoagland's nutrient solution containing 50 µm BFA for 60 min and then transferred to solution containing 5 µM FM4‐64 for 30 min. Similar segments of roots from WT, *Zmrlr1_ems_*, and *Zmrlr1* mutants were then sampled and transferred to glass slides. Internalization of FM4‐64 was monitored with three‐dimensional reconstructions of z‐stacks at the indicated durations using a Zeiss LSM900 confocal microscope.

### 4.13 RNA‐Seq Analysis

Total RNA was extracted from the roots of WT maize, *Zmrlr1#1* mutants, and *ZmRLR1*‐overexpressing transgenic maize (*#1*). Approximately 3 µg of total RNA was used as input material for the construction of RNA libraries. Transcriptome sequencing was performed using the BGI DNBSEQ‐T7 high‐throughput sequencing platform by the Hainan Huada Gene Technology Co., Ltd. (Sanya, China).

The raw reads were trimmed using fastp [58] and aligned to maize B73_RefGen_v4 (ftp://ftp.ensemblgenomes.org/pub/plants/release‐41/fasta/zea_mays/dna/) using HISAT2 v2.1.0 with default parameters [59]. Only perfectly matching sequences were retained for further analysis. Normalized gene expression levels were determined by the count information of per kilobase per million mapped reads [60]. DEGs were identified by Feature Counts software and the R package “edgeR” [61]. Genes were considered as DEGs if the log_2_ fold‐change ratio was ≥ 1 and the *p* value was < 0.05. The GO results were analyzed by TBtools [62].

### 4.14 Candidate Gene‐Based Association Analysis

A total of 122 maize inbred lines were used for the candidate gene‐based association analysis. The promoter and coding regions of *ZmRLR1* were generated through targeted resequencing technology. The sequencing process and analysis pipeline were performed as previously described [63]. Clean reads were mapped to *ZmRLR1* using BWA v0.07.17 [64]. Individual VCF calling, population SNP calling, and InDel calling were performed according to the Genome Analysis Toolkit (GATK, v.4.2.6.1, U.S.A.) [65]. For SNP filtering, VariantFiltration was employed to exclude potential false‐positive variant calls based on the following parameters: QUAL < 30.0, FS > 60.0, QD < 2.0, SOR > 3.0, MQ < 40.0, MQRankSum < −12.5, and ReadPosRankSum < −8.0. Candidate gene‐based association analysis was performed using TASSEL v.5.0 with a general linear model [66]. The linkage disequilibrium plot was generated in HAPLOVIEW [67].

### 4.15 Analysis of RSA

To assess lodging events in maize at the tasseling stage, the degree of root lodging of the inbred lines was immediately investigated on the day following a violent rainstorm. The severity of root lodging was assessed by measuring the angle between the stem and the ground. The severity was divided into six classes: I (angle ≤ 15°), II (15° < angle ≤ 30°), III (30°< angle ≤ 45°), IV (45° < angle ≤ 60°), V (60°< angle ≤ 75°), and VI (75° < angle ≤ 90°).

For root RSA analysis, WT, *Zmrlr1_ems_*, *Zmrlr1* mutants, and *ZmRLR1*‐overexpressing transgenic maize were cultivated in 21‐L soil pots until they reached the V12 stage. The inbred lines were planted in rhizoboxes to investigate root morphology at V3 stage. The roots were initially soaked in water to loosen the adhering soil. Subsequently, the remaining soil was carefully removed using a washing apparatus equipped with adjustable water pressure. Hydroponically grown WT, *Zmrlr1_ems_*, *Zmrlr1* mutants, and *ZmRLR1*‐overexpressing transgenic maize at the V4 stage were also selected for RSA analysis. The roots were placed in a transparent tray. Root images were obtained using a flatbed scanner (Epson Perfection V850 Pro, Japan) at a resolution of 400 dpi. Images were analyzed with Win‐RHIZO Pro 2019 (Instruments Regent Inc., Canada).

To quantify root primordia, seminal roots devoid of lateral root emergence were incubated in a solution of ethanol and glacial acetic acid (3:1 v/v) at 4°C for 24 h. After being washed with deionized water, the roots were transferred to a 50% sodium hypochlorite solution for 10 min. The roots were subsequently incubated in 0.01% methylene blue for 30 min. After overnight decolorization with 10% glycerin at 4°C, lateral root primordia were captured with an Asana mirror (OLYMPUS SZX2‐ILLK, Japan).

### 4.16 IAA Analysis

Roots of hydroponic WT and *Zmrlr1* mutants at the V3 stage were sampled for free IAA analysis. Free IAA extraction, purification, and quantification were performed as previously described [45]. Each experiment was replicated three times.

The *ZmPIN1a*‐YFP and *ZmDR5*‐mRFP lines in the B73 background were generously provided by Dr. Fang Yang from Sun Yat‐sen University. These lines were crossed with *Zmrlr1_ems_*. The resulting *Zmrlr1_ems_* plants harboring the *DR5‐RFP* or *PIN1a‐YFP* reporter genes were selected from the BC_3_F_2_ populations. The seminal roots of *Zmrlr1_ems_* plants harboring the *DR5‐RFP* or *PIN1a‐YFP* reporter genes were used to observe RFP and YFP signals. The signals were collected using a Zeiss LSM 980 confocal microscope.

### 4.17 Determination of AsA and H_2_O_2_ Contents

Roots of hydroponic WT, *Zmrlr1_ems_*, *Zmrlr1* mutants, and *ZmRLR1*‐overexpressing transgenic maize at the V3 stage were sampled for AsA and H_2_O_2_ analysis. The AsA and H_2_O_2_ contents were determined according to the manufacturer's procedures (Beijing Solarbio Technology Co., Ltd., China).

### 4.18 Measurement of Stem Pulling Force

The stem pulling force of field‐grown maize at the tasseling stage was measured using a plant lodging resistance tester (TPDF‐1, Zhejiang Top Cloud‐Agri Technology Co., China). The measurements were performed at a height of 50 cm above the ground with a pulling angle of 15° (Figure S16). Twenty plants of each genotype were measured.

### 4.19 Statistical Analyses

Statistical significance between two groups was assessed using the Student's *t*‐test. For comparisons of multiple groups, the data were analyzed by one‐way analysis of variance (ANOVA), followed by Tukey's multiple comparison test using SAS (v.8; SAS Institute Inc., Cary, NC, U.S.A.). IBM SPSS Statistics 21 (IBM Corp., U.S.A.) was used for Pearson correlation analysis.

## Author Contributions

WXL and QD designed the research; WL, PY, HC, GL and YZ performed the research; QD, WXL and YC analyzed the data; WXL wrote the article.

## Conflicts of Interest

The authors declare no conflicts of interest.

## Supporting information




**Supporting File 1**: advs73781‐sup‐0001‐FigureS1‐S16.pdf.

**Supporting File 2**: advs73781‐sup‐0002‐TableS1.xlsx.

**Supporting File 3**: advs73781‐sup‐0003‐TableS2.xlsx.

**Supporting File 4**: advs73781‐sup‐0004‐TableS3.xlsx.

**Supporting File 5**: advs73781‐sup‐0005‐Figure 8originaldata.zip.

## Data Availability

The RNA‐seq data generated from this research have been deposited in the National Genomics Data Center (https://ngdc.cncb.ac.cn/) under accession number CRA017886.
