# Supplementary material for: Natural Variations of ZmRLR1 Mediate the Root Lodging Resistance of Maize by Regulating Root Ascorbate and Auxin Homeostasis
Source: Adv Sci (Weinh). 2026 Jan 15;13(17):e19638. doi: 10.1002/advs.202519638 (PMC13042799; doi:10.1002/advs.202519638)
Supplement: Supplementary file 1 — Supporting File 1: advs73781‐sup‐0001‐FigureS1‐S16.pdf. [file ADVS-13-e19638-s004.pdf]

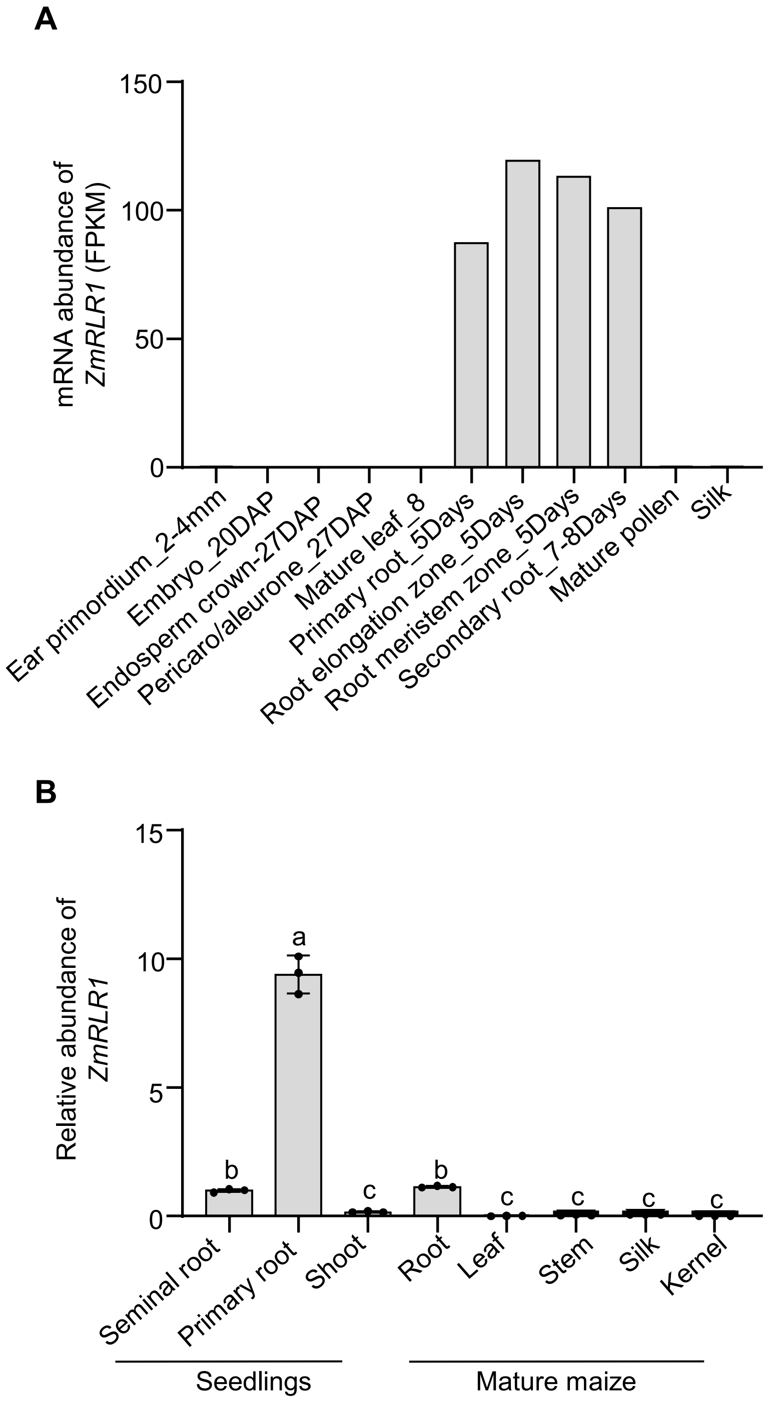

Figure S1. Expression patterns of *ZmRLR1* in maize. (A) Analysis of *ZmRLR1* expression data downloaded from the National Center for Biotechnology Information. (B) The mRNA abundance of *ZmRLR1* in various tissues of maize as determined by RT-qPCR. The data were normalized to the expression of *ZmUBQ1*. The error bars represent the standard deviations of three biological replicates. Means with the same letter are not significantly different at $P < 0.05$ according to one-way ANOVA followed by Tukey's multiple comparison test.

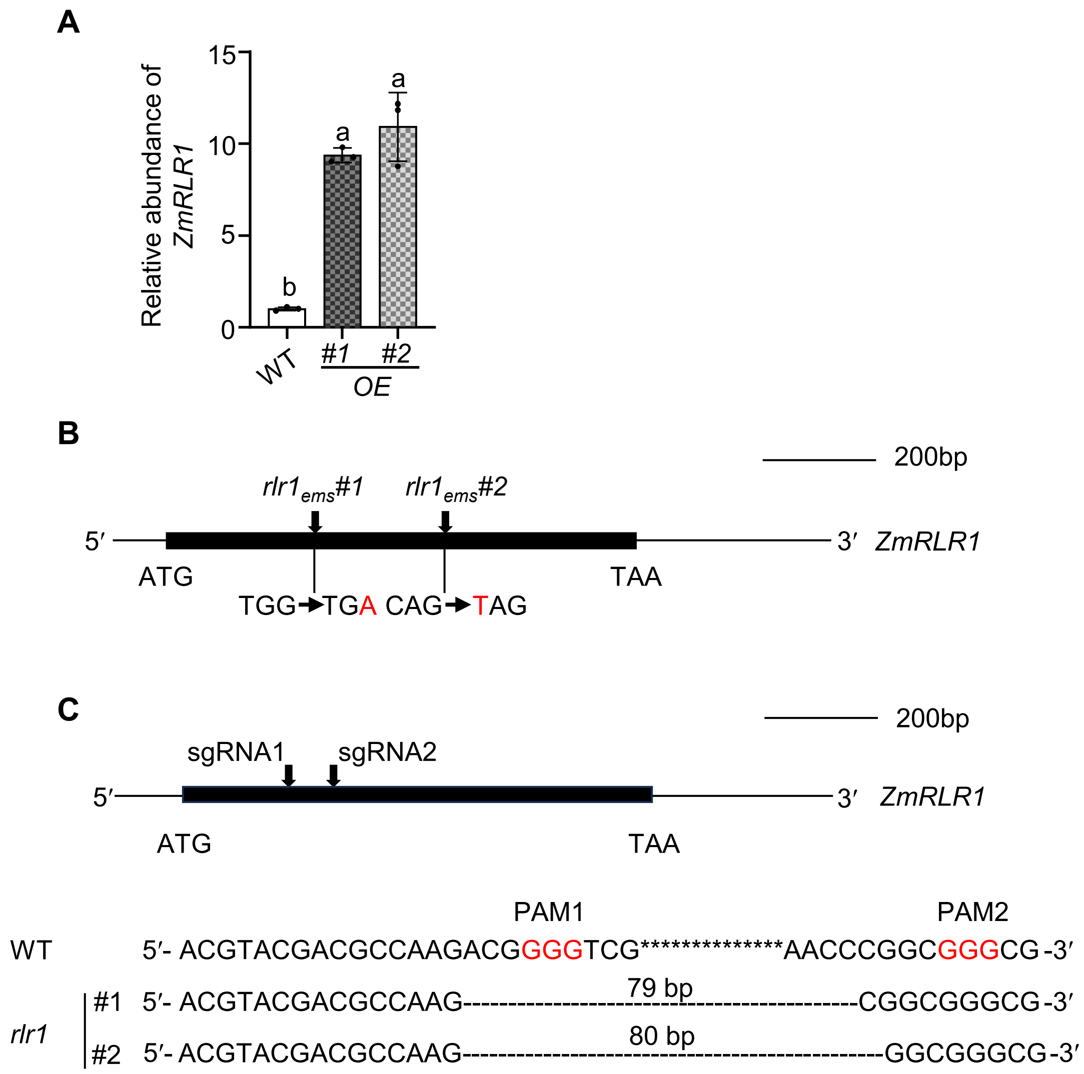

Figure S2. Identification of *ZmRLR1*-overexpressing transgenic maize and *ZmRLR1* loss-of-function mutants. (A) Relative expression levels of *ZmRLR1* in wild type (WT) and *ZmRLR1*-overexpressing transgenic maize (*OE*). The data were normalized to the expression of *ZmUBQ1*. The error bars represent the standard deviations of three biological replicates. Means with the same letter are not significantly different at $P < 0.05$ according to one-way ANOVA followed by Tukey's multiple comparison test. (B) Diagram of the EMS-mutated sites in *ZmRLR1*. (C) Diagram of synthetic guide RNAs (sgRNAs) and the observed targeted deletion mutations of *ZmRLR1* between the two sgRNA-mediated sites.

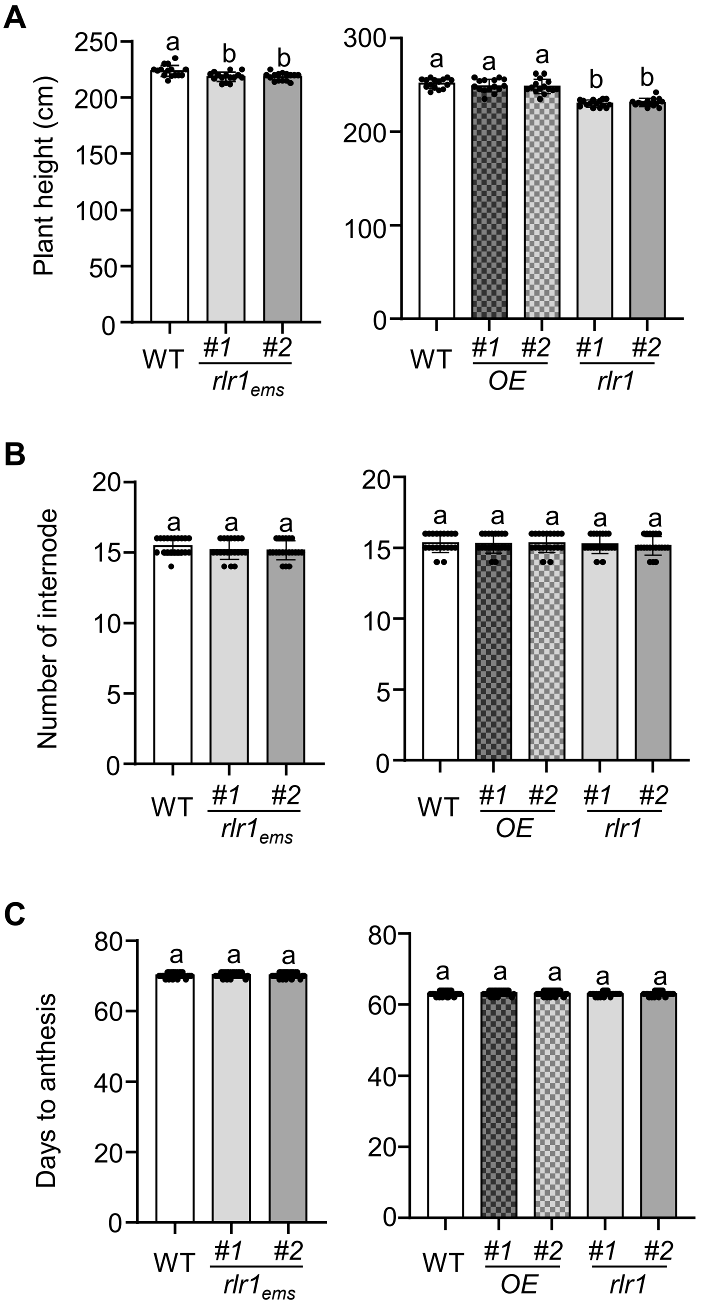

Figure S3. Agronomic traits of *Zmrlr1*$_{ems}$, *Zmrlr1* mutants, and *ZmRLR1*-overexpressing transgenic maize (*OE*). (A) Effects of *ZmRLR1* expression on the height of maize (n = 15). (B) Effects of *ZmRLR1* expression on the internode number of maize (n = 20). (C) Effects of *ZmRLR1* expression on the days to anthesis of maize (n = 30). Means with the same letter are not significantly different at $P < 0.05$ according to one-way ANOVA followed by Tukey's multiple comparison test.

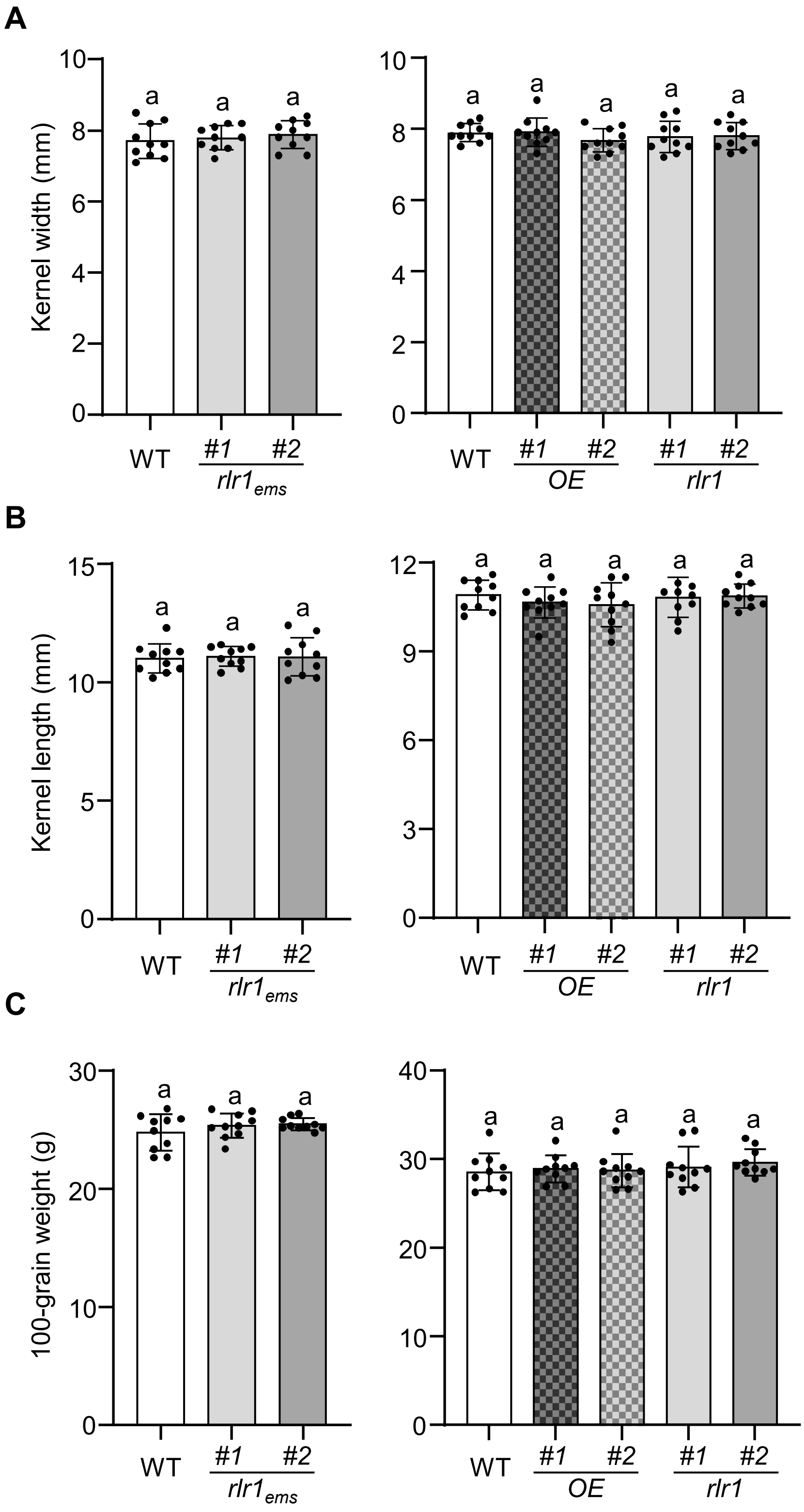

Figure S4. *ZmRLR1* has no detectable impact on maize kernel traits. (A) Effects of *ZmRLR1* expression on the kernel width of maize (n = 10). (B) Effects of *ZmRLR1* expression on the kernel length of maize (n = 10). (C) Effects of *ZmRLR1* expression on the 100-kernel weight of maize (n = 10). Means with the same letter are not significantly different at $P < 0.05$ according to one-way ANOVA followed by Tukey's multiple comparison test.

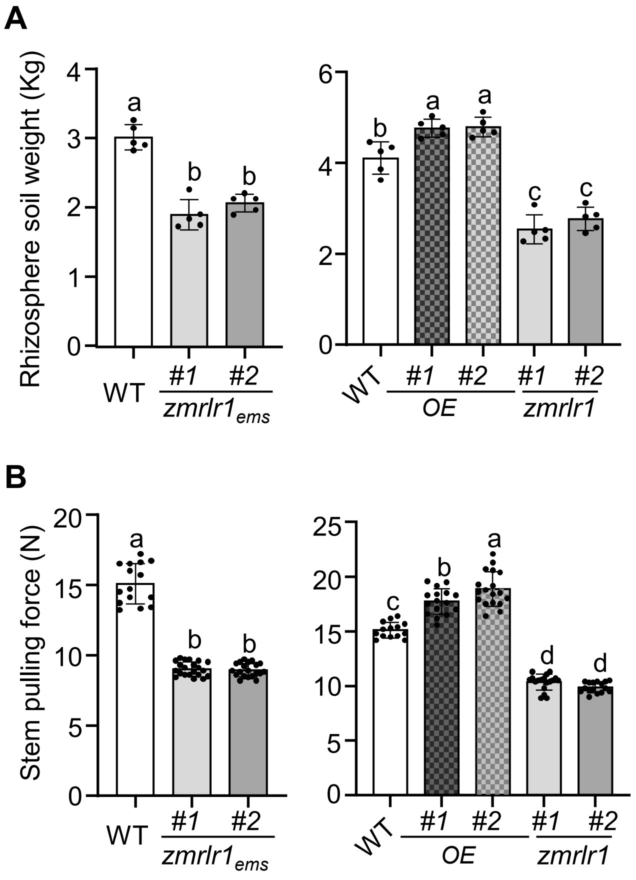

Figure S5. *ZmRLR1* positively regulates the root lodging resistance of maize in Sanya, Hainan Province. (A) Effects of *ZmRLR1* expression on the rhizosphere soil weight of maize (n = 5). (B) Effects of *ZmRLR1* expression on the stem pulling force of the internode 50 cm from the ground (n = 14-20). Means with the same letter are not significantly different at $P < 0.05$ according to one-way ANOVA followed by Tukey's multiple comparison test.

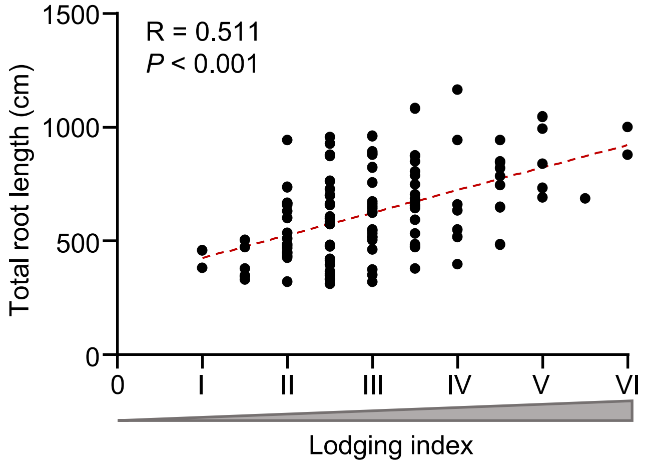

Figure S6. Pearson's correlation coefficient between the total root length and lodging index.

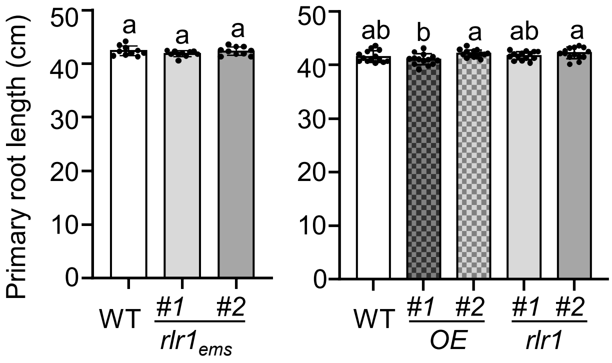

Figure S7. *ZmRLR1* does not affect primary root length. n = 10-14. Means with the same letter are not significantly different at $P < 0.05$ according to one-way ANOVA followed by Tukey's multiple comparison test.

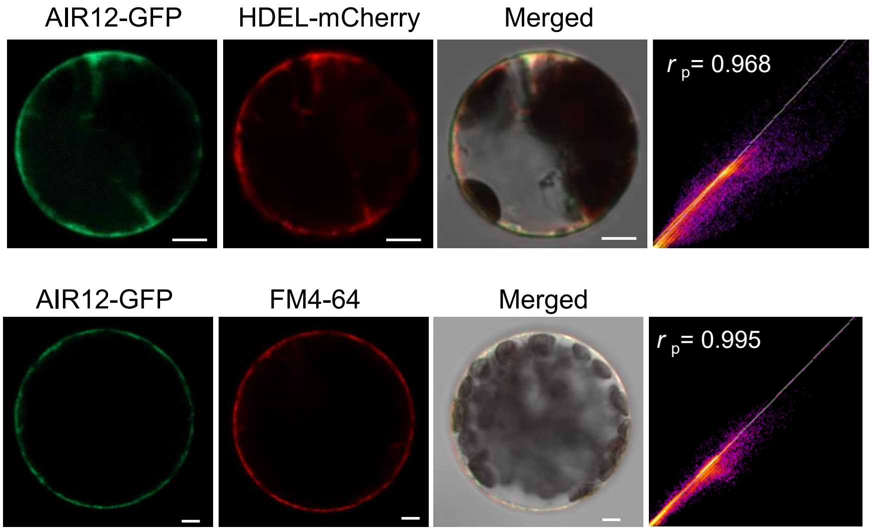

Figure S8. Subcellular localization of the AtAIR12 protein. HDEL-mCherry and FM4-64 were used as endoplasmic reticulum and plasma membrane markers, respectively. Scale bars = 5 μm.

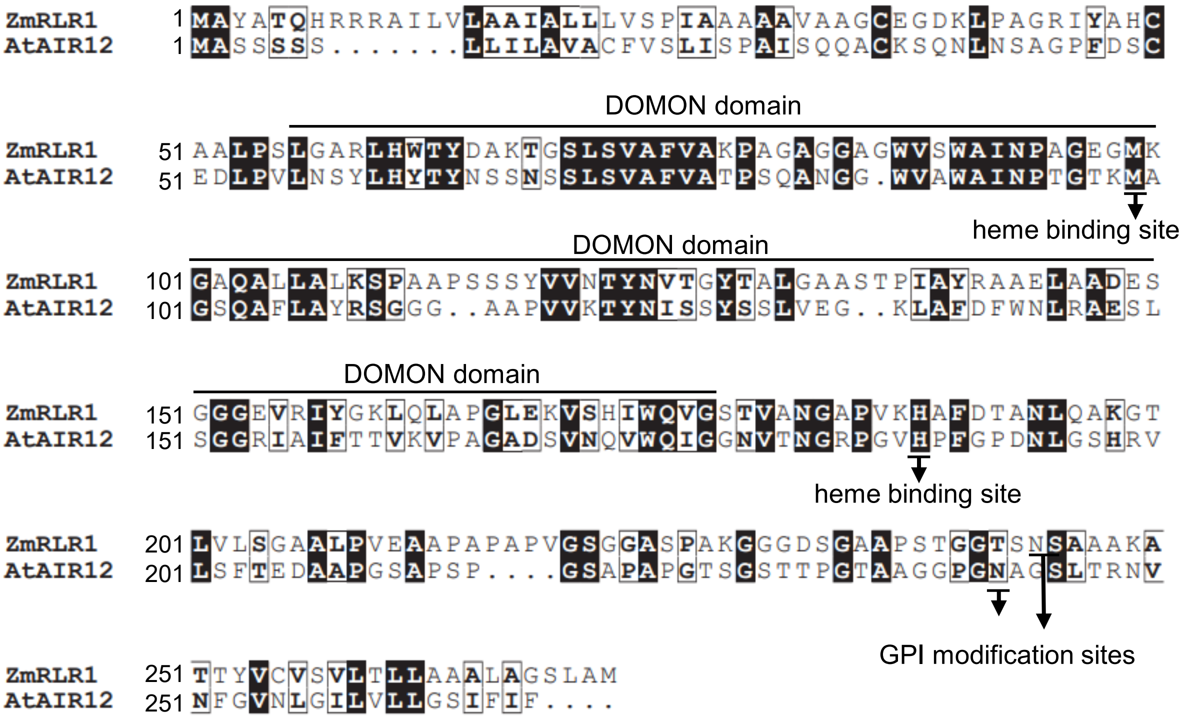

Figure S9. The alignment of ZmRLR1 and AtAIR12. The motifs are underlined.

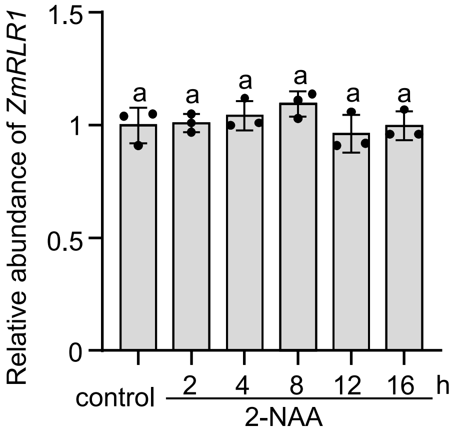

Figure S10. Effects of 2-NAA on the mRNA abundance of *ZmRLR1*. The data were normalized to the expression of *ZmACTIN1.* The error bars represent the standard deviations of three biological replicates. Means with the same letter are not significantly different at $P < 0.05$ according to one-way ANOVA followed by Tukey's multiple comparison test.

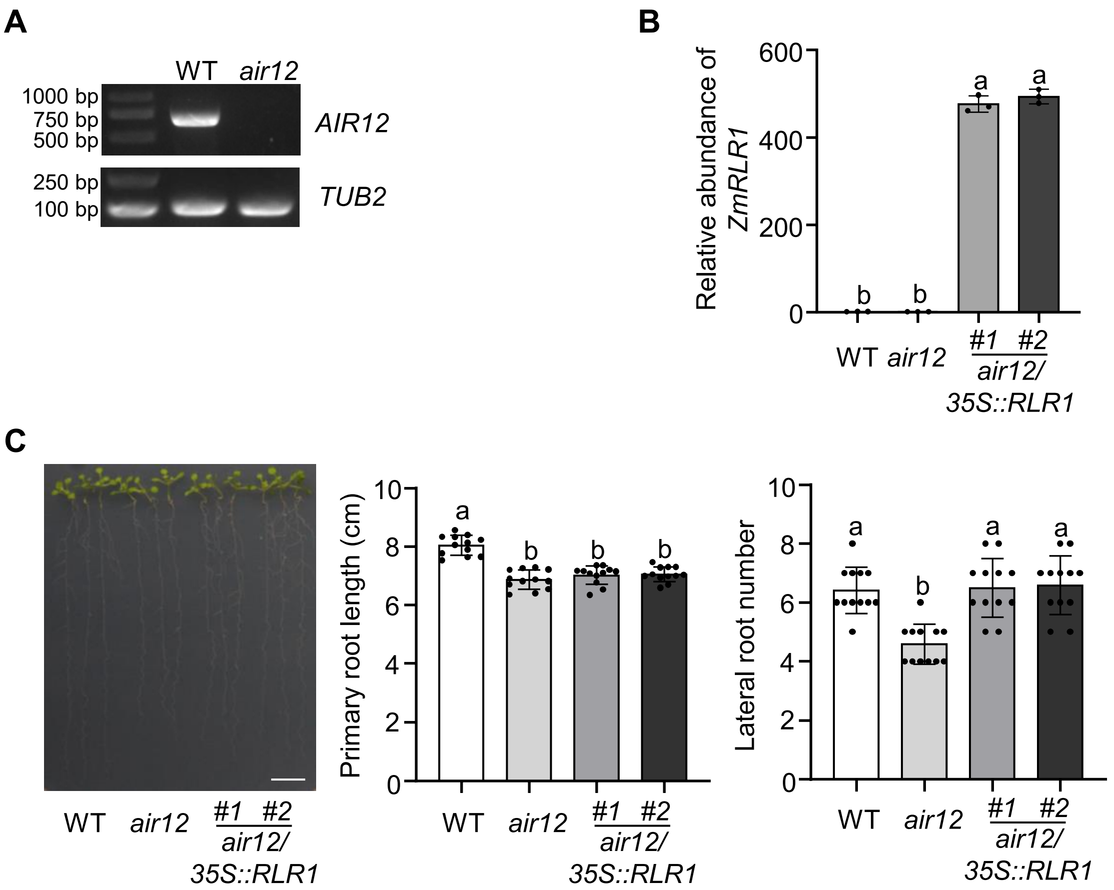

Figure S11. *ZmRLR1* can partially rescue root development defects in *Atair12* mutant. (A) Detection of *AIR12* mRNA in *Atair12* mutant by RT-PCR. *AtTUB2* was used as the loading control. (B) Relative expression levels of *ZmRLR1* in *Atair12* mutant. The data were normalized to the expression of *AtTUB2*. (C) Effects of *ZmRLR1* expression on the primary root length and the number of lateral root of *Arabidopsis* (n = 12). Scale bar = 1 cm. Seedlings were grown on MS agar medium for 12 d. Means with the same letter are not significantly different at $P < 0.05$ according to one-way ANOVA followed by Tukey's multiple comparison test.

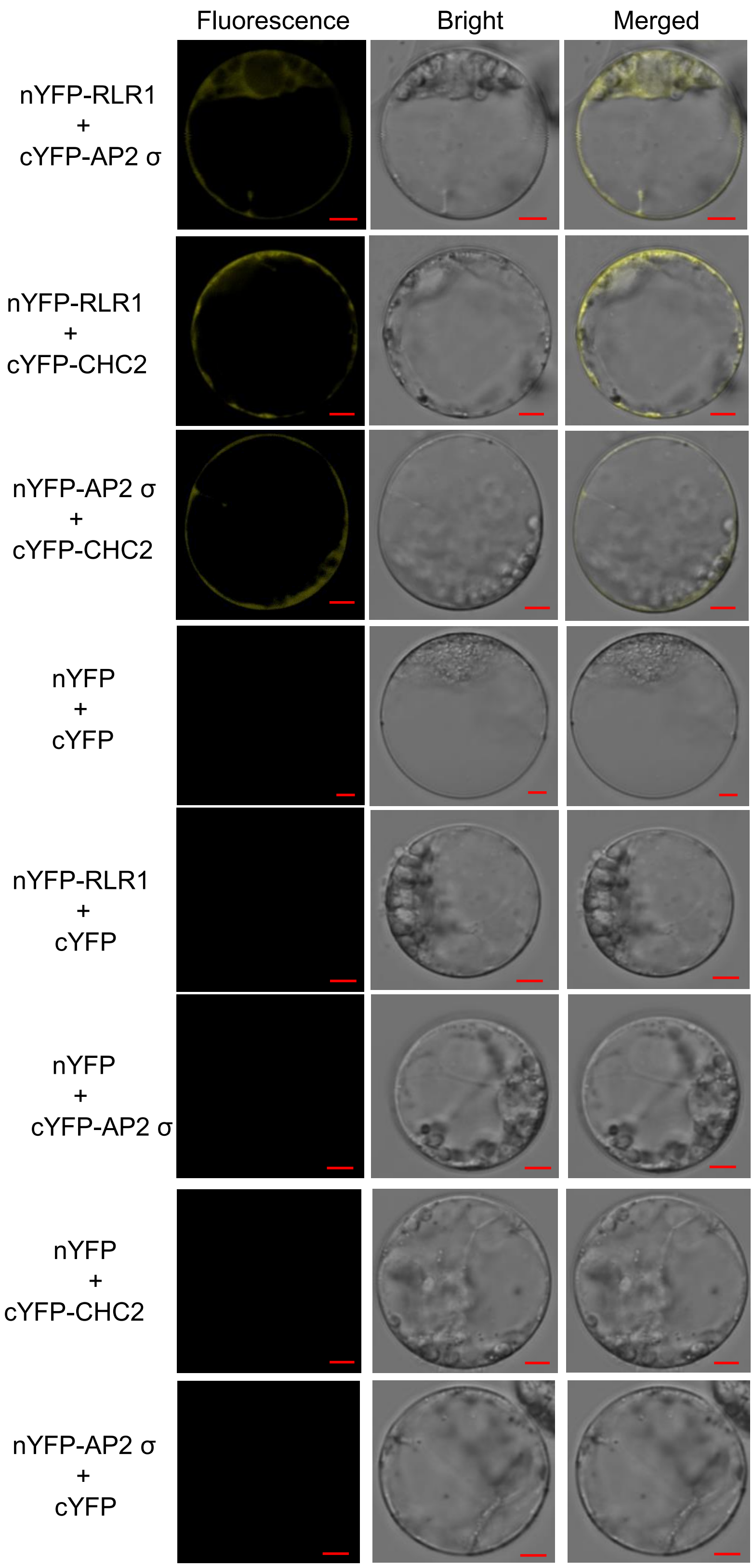

Figure S12. Interactions between ZmRLR1 and the ZmAP2 σ subunit, ZmRLR1 and ZmCHC2, and the ZmAP2 σ subunit and ZmCHC2 in maize mesophyll protoplast observed by BiFC assays. Scale bars = 5 μm.

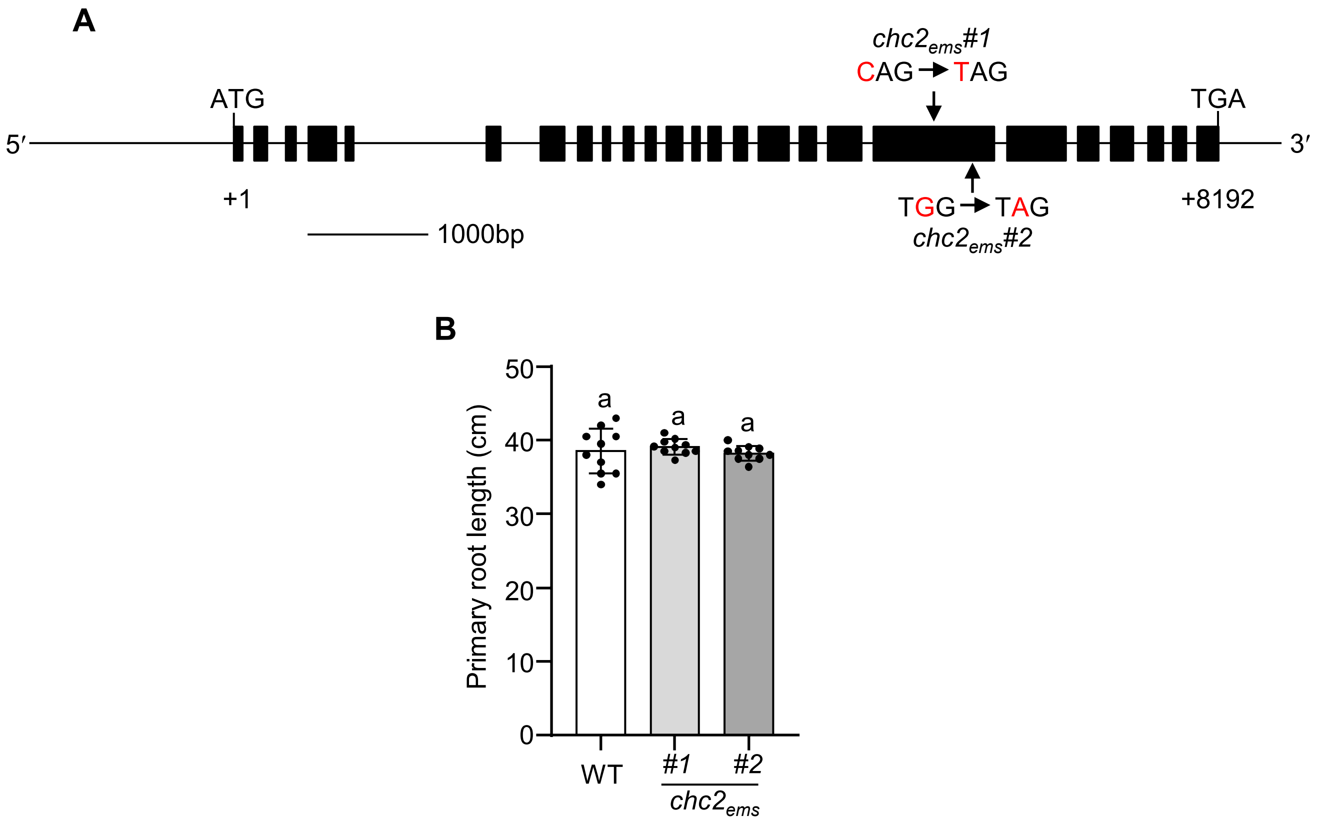

Figure S13. Diagram of the EMS-mutated sites in *ZmCHC2* (A) and the primary root length of *Zmchc2*$_{ems}$ (B). *n* = 10. Means with the same letter are not significantly different at $P < 0.05$ according to one-way ANOVA followed by Tukey's multiple comparison test.

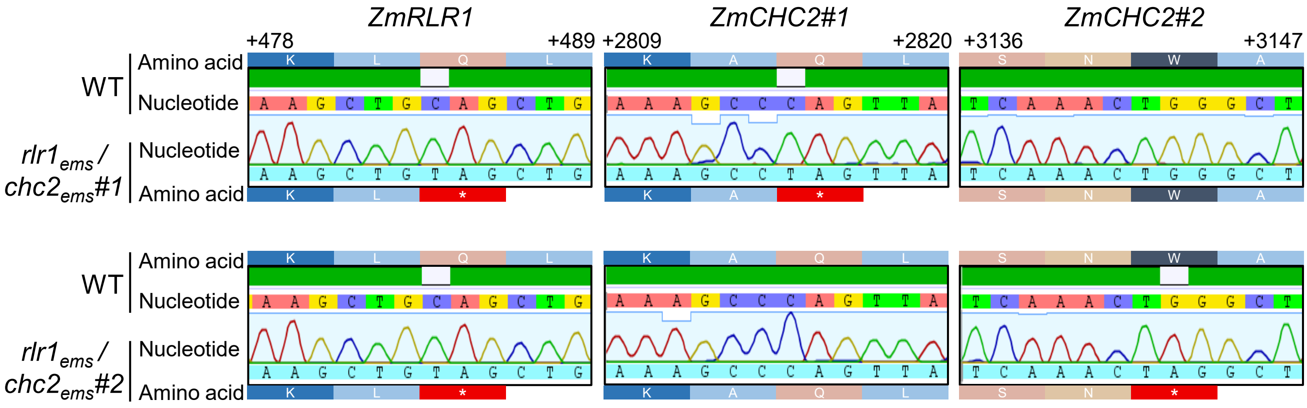

Figure S14. Identification of the double mutant.

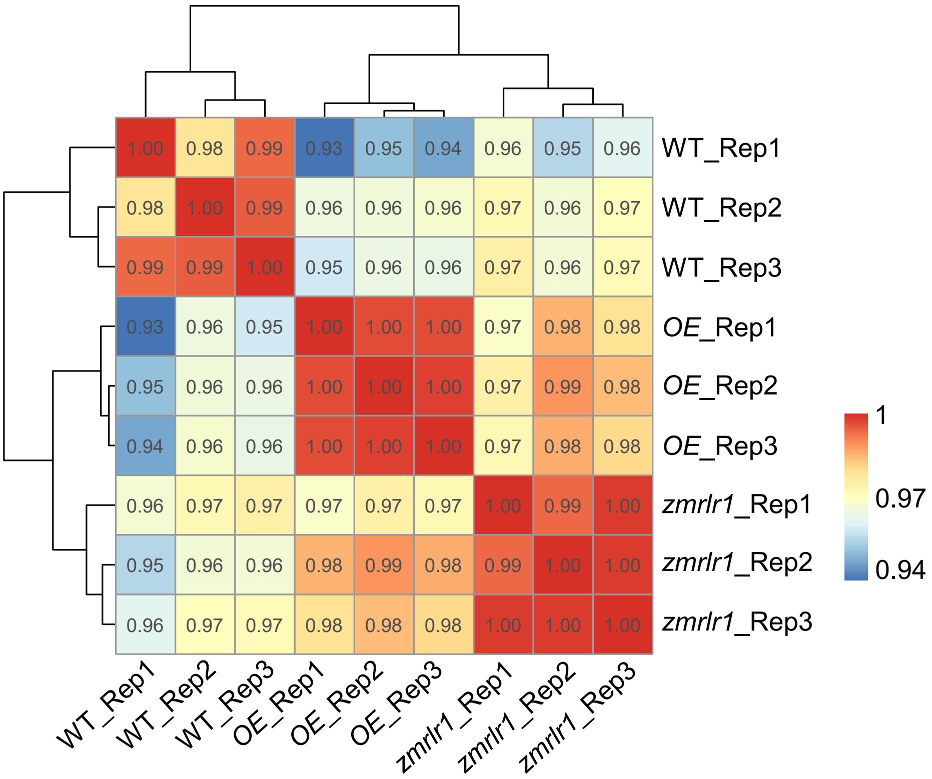

Figure S15. Correlation matrix of RNA-seq biological replicates of WT, *Zmrlr1* mutant, and *ZmRLR1*-overexpressing transgenic maize (*OE*). The color bar represents the Pearson's correlation coefficient from 0.94 (blue) to 1 (red).

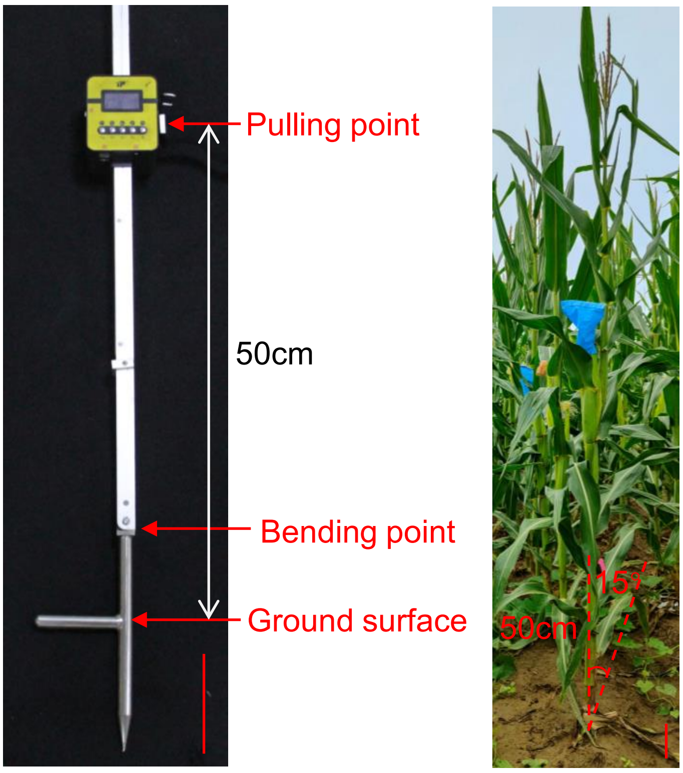

Figure S16. Schematic diagram of stem pulling force measurement. Scale bars = 10 cm.
