# Supplementary figures and images for: Natural Variations of ZmRLR1 Mediate the Root Lodging Resistance of Maize by Regulating Root Ascorbate and Auxin Homeostasis
Source: Adv Sci (Weinh). 2026 Jan 15;13(17):e19638. doi: 10.1002/advs.202519638 (PMC13042799; doi:10.1002/advs.202519638)

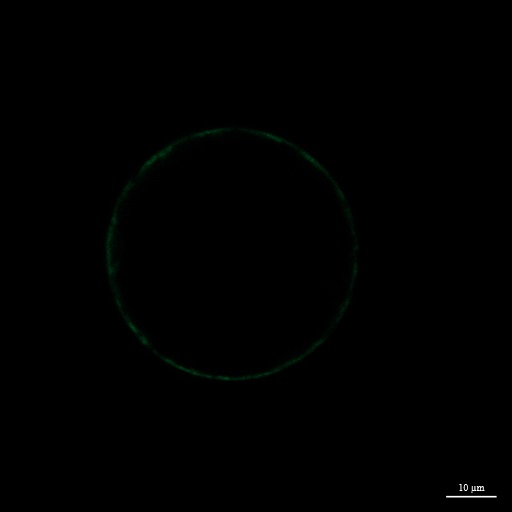

Supplement: Supplementary file 5 — Supporting File 5: advs73781‐sup‐0005‐Figure 8originaldata.zip. [file ADVS-13-e19638-s002.zip › AIR12-GFP.jpg]

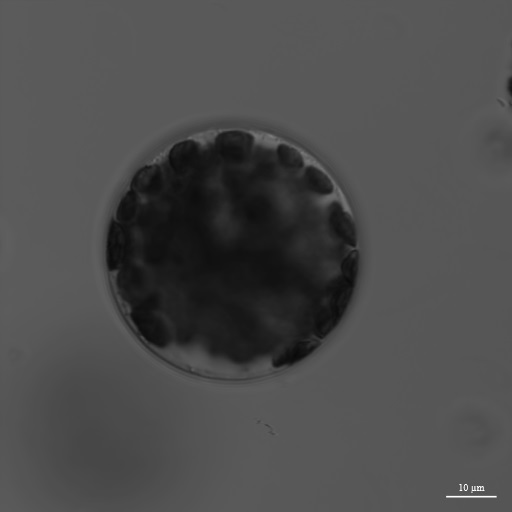

Supplement: Supplementary file 5 — Supporting File 5: advs73781‐sup‐0005‐Figure 8originaldata.zip. [file ADVS-13-e19638-s002.zip › Bright.jpg]

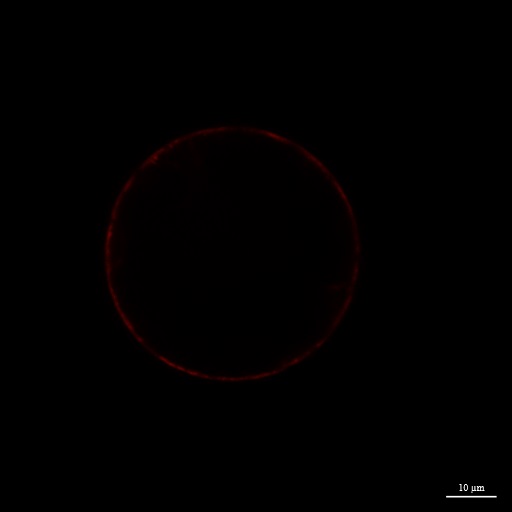

Supplement: Supplementary file 5 — Supporting File 5: advs73781‐sup‐0005‐Figure 8originaldata.zip. [file ADVS-13-e19638-s002.zip › FM4-64.jpg]

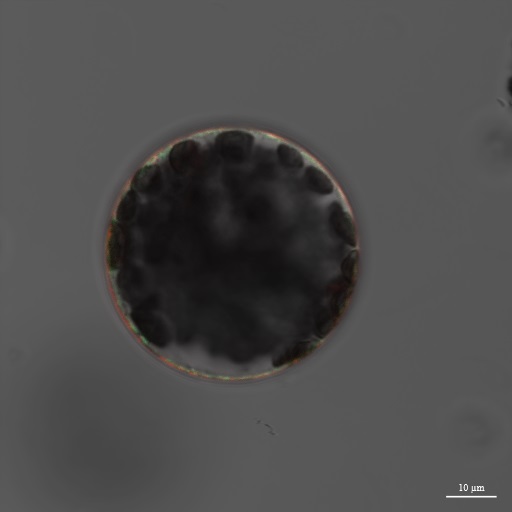

Supplement: Supplementary file 5 — Supporting File 5: advs73781‐sup‐0005‐Figure 8originaldata.zip. [file ADVS-13-e19638-s002.zip › Merged.jpg]
